# Exploratory Synthetic Studies of the Praseodymium/Di-2-Pyridyl Ketoxime System Leads to Unusual Reactivity and Interesting New Molecules

**DOI:** 10.1021/acs.inorgchem.5c04100

**Published:** 2025-12-12

**Authors:** Christina Stamou, Christina Polyzou, Constantinos C. Stoumpos, Catherine P. Raptopoulou, Dionissios Papaioannou, Yiannis Sanakis, Vassilis Psycharis, Spyros P. Perlepes

**Affiliations:** † Department of Chemistry, 37795 University of Patras, 26504 Patras, Greece; ‡ Institute of Nanoscience and Nanotechnology, NCSR “Demokritos”, 15310 Aghia Paraskevi Attikis, Greece; § Institute of Chemical Engineering Sciences, Foundation for Research and Technology-Hellas (FORTH/ICE-HT), Platani, P.O. Box 1414, 26504 Patras, Greece

## Abstract

Reactions of lanthanoid­(III) ions with di-2-pyridyl ketoxime, dpkoxH, have been studied. Full synthetic investigation of the Pr­(NO_3_)_3_·6H_2_O/dpkoxH reaction system has provided access to complexes [Pr_2_^III^(NO_3_)_4_(L)_2_] (isolated as MeCN and MeNO_2_ solvates; **1** and **2**), [Pr_4_^III^(OH)_2_(NO_3_)_4_(dpkox)_6_(EtOH)_2_] (**3**) and [Pr_8_^III^Pr^IV^O_4_(OH)_4_(NO_3_)_4_(dpkox)_12_(H_2_O)_4_] (**4**). A novel Pr^III^-assisted/promoted ligand transformation has occurred in the dinuclear complexes, where L^–^ is the anion of di­(pyridin-2-yl)­methanone *O*-(1-hydroxy-1,1-di­(pyridin-2-yl)) methyl oxime (HL). Mechanistic schemes have been proposed. In **1** and **2**, the two Pr^III^ centers are bridged by two deprotonated oxygen atoms of two “head-to-head” 2.2011110 (Harris notation) L^–^ ligands. The tetranuclear molecule **3** is held together by two μ_3_-hydroxido groups, two 2.2110, two 2.1110 and two 3.2110 dpkox^–^ ligands. The {Pr_4_^III^(μ_3_–OH)_2_}^10+^ unit of the {Pr_4_^III^(μ_3_–OH)_2_(μ_2_-oximato)_4_}^6+^ core comprises four coplanar Pr^III^ ions in a “butterfly”-type disposition. In the highly symmetrical cluster **4**, the nine Pr ions are held together by four μ_3_-O^2–^ and four μ_3_–OH^–^ groups, as well as four 2.1110, four 3.2111 and four 2.2110 dpkox^–^ ligands. The inorganic {Pr_8_^III^Pr^IV^(μ_3_-O)_4_(μ_3_–OH)_4_}^16+^ unit of the core can be described as four fused “butterflies”, the Pr^IV^ center being the common site at one of their “body” positions. The X-band (∼9.5 GHz) EPR spectrum of **4** at 4.2 K shows a sharp peak at 630 G, a derivative feature at 1340 G, and a multitude of weaker signals; the data are compatible with the presence of the half-integer spin expected for the 4f^1^ Pr^IV^ center.

## Graphical abstract

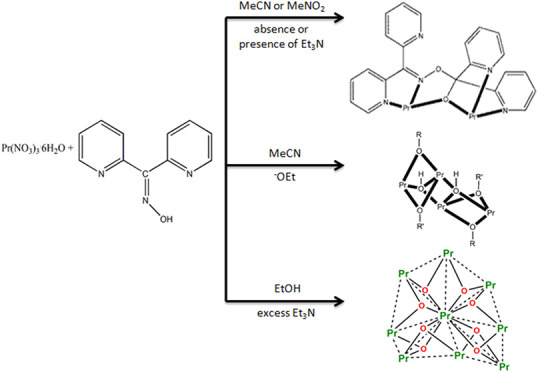

## Introduction

The on purpose utilization of known ligands and the synthesis of new ones are behind of many developments in inorganic, bioinorganic and metallosupramolecular chemistry. Concepts related to ligands are the chelate, macrocyclic and cryptate effects, chirality, isolobal relationships, the conformation of chelating rings and the reactivity of coordinated ligands.^1^

The oxime moiety (R_1_R_2_C=NOH) is an important functional group in organic chemistry^2,3^ but it also plays an important role in inorganic,^4,5^ supramolecular,^6,7^ industrial^8^ and medicinal chemistry.^9^ Interestingly the first application of oximes was in the field of analytical chemistry; in 1885, Tschugaeff used dimethylglyoxime for the classical gravimetric determination of Ni^2+^ exploiting the insolubility of bis­(dimethylglyoximato)­nickel­(II).^10^ In inorganic chemistry, the oxime group is most often part of a ligand that possesses one or more donor sites.^11^ One category of such ligands is the family of 2-pyridyl oximes (Chart 1, left), where R is a nondonor site. These ligands are popular because of their relationship to several areas, including molecular magnetism (synthesis of single-molecule magnets, SMMs,^12^ and single-chain magnets, SCMs^13^), preparation of 3d/4f-metal compounds,^14^ understanding the molecular basis of solvent extraction of toxic metal ions from aqueous media^15^ and linking of low-nuclearity clusters to higher-nuclearity assemblies with the help of coordination bonds.^16^

**Chart 1:**
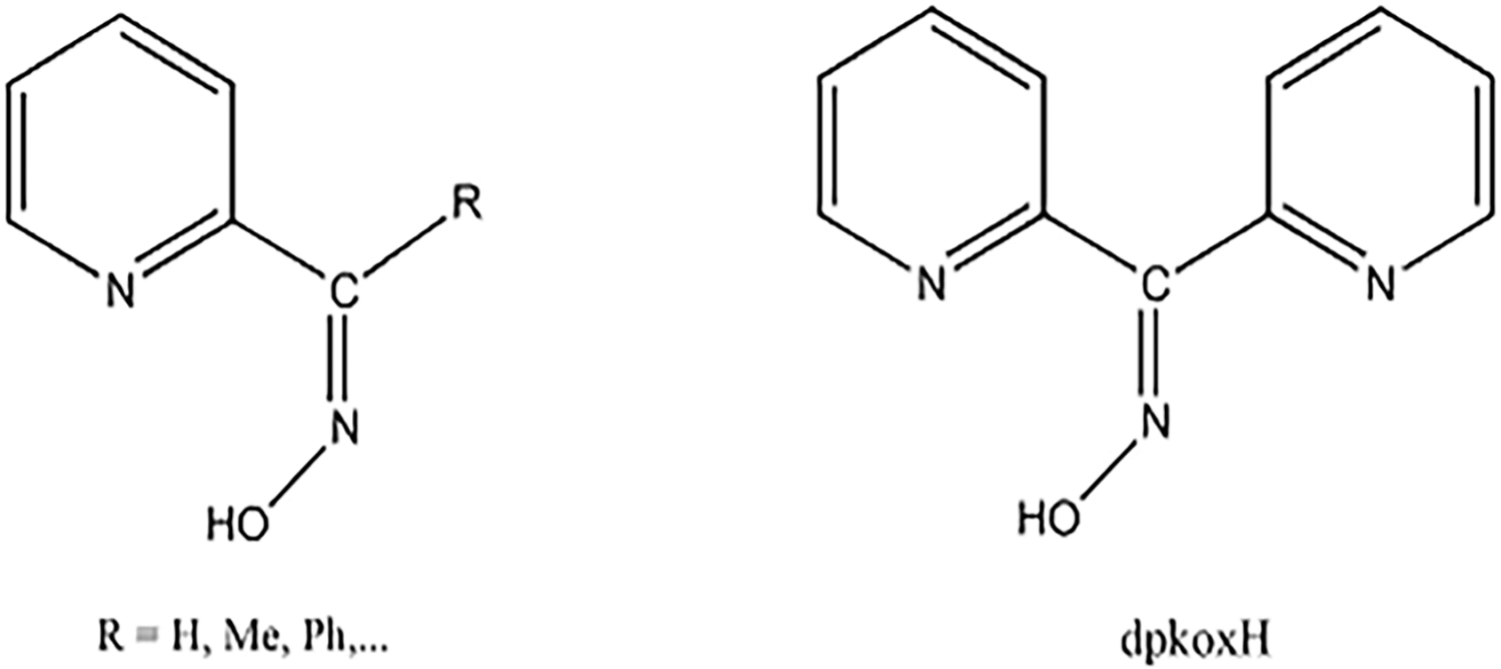
Structural Formulae of Simple 2-Pyridyl Oximes (R is a Non-Donor Site) and Di-2-Pyridyl Ketoxime (Abbreviated as dpkoxH)

When R is a donor site, 2-pyridyl ketoximes are becoming more complicated, but equally (or more) interesting. Di-2-pyridyl ketoxime (dpkoxH; Chart 1, right; its IUPAC name is di-2-pyridin-2-yl-methanone oxime) is a unique member of this family for two reasons: (i) The R donor site is a second 2-pyridyl group; and (ii) the neutral molecule and its anion (dpkox^–^) exhibit an extraordinary coordination flexibility and versatility (*vide infra*) resulting in metal complexes with aesthetically beautiful molecular structures and interesting properties. This ligand is relevant to several areas, e.g., in the chemistry of metallacrowns^17,18^ and in the activation of organic ligands by transition metal ions.^19^ The coordination chemistry of dpkoxH is rich; somewhat to our surprise, homometallic f-element (both 4f and 5f) complexes of dpkoxH and dpkox^–^ have not been reported. Over the last 20 years or so, we have been working toward the creation of a “periodic table” of metal ions, whose complexes with dpkoxH and/or dpkox^–^ ligands have been synthesized and characterized.^20^ Many “boxes” (or “squares”) of this table are filled thanks to investigations by several groups,^21–24^ including our team.^18,20,25–27^ Herein, we are glad to start the completion of the blank spaces with exclusively f-metal ions (*hetero*metallic M^II^Ln_2_^III^ complexes based on dpkox^–^, with M^II^ = Ni^II^, Cu^II^ and Pd^II^, have been reported^20,28–32^ ), by describing the coordination chemistry of dpkoxH with a representative, early lanthanoid, namely Pr. In this work, we report the preparation and structural characterization of the first Pr complexes (and complexes of any 4f-element) with dpkoxH-based ligation. We describe only complexes of one lanthanoid in order to have a satisfactorily synthetic control and good comparison between the various structural types (*vide infra*), but also because Pr­(III) can be oxidized to Pr­(IV) in “hard”-base (HSAB) coordination environments in Werner-type complexes.

There has been a renaissance in the chemistry of lanthanoids since ca. 2000;^33–35^ these elements exhibit chemical similarities as a group in the 4f series of the periodic table, but simultaneously they have varied and distinctive electronic characteristics.^34^ The latter are extremely useful and form the basis for exciting properties, e.g., magnetic,^36–39^ optical,^40–42^ multifunctional behavior,^43^ catalytic,^44^ quantum computing,^45,46^ biological,^47^ etc., unusual oxidation states,^35,48–51^ studies on metal–ligand redox cooperativity^52^ and development of sophisticated bonding concepts.^53^ As far as the metal of the present work is concerned, praseodymium is most often found in the III oxidation state. However, its [Xe]­4f^3^6s^2^ configuration makes it a candidate for creating the rare oxidation states IV (*vide infra*) and V, the latter having been reported in molecular^54,55^ and material^56,57^ chemistry. Additionally, the monovalent oxidation state, i.e., Pr­(I), has been confirmed in the borozene complex [Pr^I^(B_8_^2–^)]^−^ by using a combination of photoelectron spectroscopy and theoretical calculations.^58^

This work can be considered as a continuation of our efforts in the coordination chemistry of dpkoxH^18,20,25–27^ and the chemistry of lanthanoids,^59–68^ being an amalgamation of these two areas.

## Experimental Section

### Chemicals

Praseodymium­(III) nitrate, di-2-pyridyl ketoxime and other chemicals were purchased from Sigma-Aldrich. The purity of dpkoxH was confirmed by ^1^H NMR spectroscopy in *d*_6_-DMSO. Solvents were received from various sources and used without further purification. All manipulations were performed under aerobic conditions. **Caution!** *Nitromethane is an extremely flammable and explosive liquid, which can detonate upon extreme heat. Contact with amines, alkali metals, and strong reducing agents should be strictly avoided. Di-*2*-pyridyl ketoxime and praseodymium­(III) nitrate hexahydrate cause skin, eye and respiratory irritation; breathing should be avoided, and use of protective gloves and eye laboratory glasses is recommended.*

### Instrumentation

Elemental analyses (C, H, N) were performed by the Instrumental Analysis Service of the University of Patras using a PerkinElmer 2400 analyzer. Fourier transform infrared (FT-IR) spectra were recorded using a PerkinElmer 16PC spectrometer; the samples were in the form of KBr pellets (prepared under pressure), and nujol or hexachlorobutadiene mulls between CsI disks. The ^1^H NMR spectrum of the free ligand was recorded on a 600.13 MHz Bruker Avance DPX spectrometer. X-band EPR measurements were carried out on an upgraded Bruker ER-200D spectrometer equipped with an Oxford ESR 900 cryostat, an Anritsu MF76A frequency counter, and a Bruker 035 M NMR gaussmeter.

**Chart 2:**
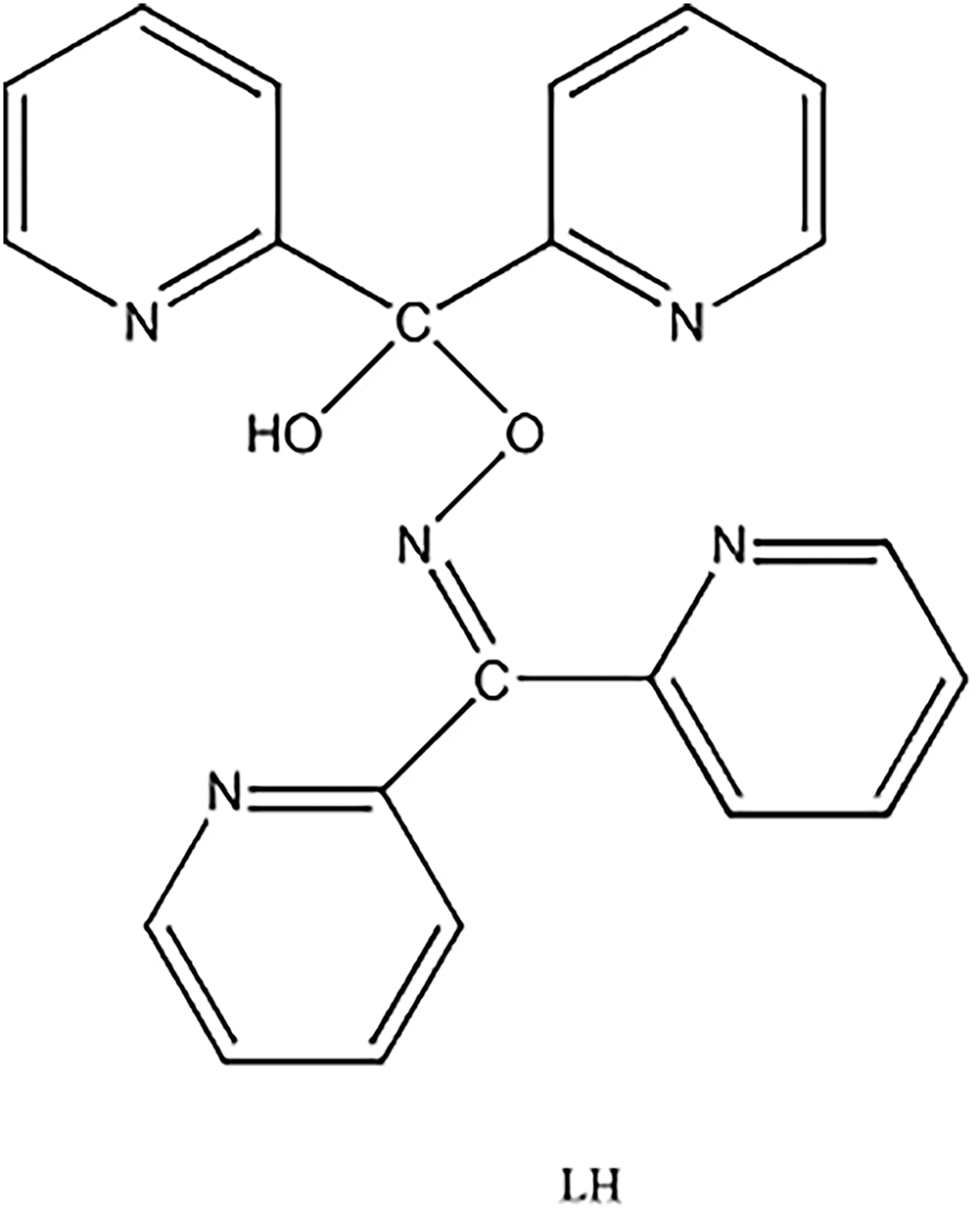
Structural Formula of Di­(pyridin-2-yl)­methanone *O*-(1-Hydroxy-1,1-di­(pyridin-2-yl)) Oxime (Abbreviated as LH)

#### Synthesis of [Pr_2_(NO_3_)_4_(L)_2_]·3MeCN (1·3MeCN), Where L^–^ is the Anion of Di­(pyridin-2-yl)­methanone *O*-(1-Hydroxy-1,1-di­(pyridin-2-yl))­methyl Oxime (Chart 2)

##### Method A

Solid dpkoxH (0.080 g, 0.40 mmol) was added to a stirred pale green solution of Pr­(NO_3_)_3_·6H_2_O (0.174 g, 0.40 mmol) in MeCN (25 mL). The resulting green solution was stirred for a further 30 min, filtered, and stored in a flask at room temperature. X-ray quality, pale green crystals of the product were precipitated in a period of 6 weeks. The crystals were collected by filtration, washed with cold MeCN (2 mL) and Et_2_O (2 × 3 mL), and dried in a vacuum desiccator over silica gel. The yield was ∼20% (based on the Pr^III^ available). The complex was analyzed satisfactorily as lattice solvent-free, i.e., as **1**. Anal. Calcd for C_44_H_32_Pr_2_N_14_O_16_ (found values in parentheses): C 40.82% (40.47%); H 2.50% (2.59%), and N 15.15% (14.91%). Selected IR data (KBr, cm^–1^): 1594­(m), 1470(s), 1438­(m), 1384(s), 1282(s), 1004(s), 798­(m), 752­(m), 676­(m), 628­(m), 586­(w).

##### Method B

To a pale green solution containing Pr­(NO_3_)_3_·6H_2_O (0.435 g, 1.00 mmol) and dpkoxH (0.199 g, 1.00 mmol) in MeCN (10 mL) was added slowly Et_3_N (0.14 mL, 1.00 mmol) under stirring. The resulting green solution was stirred for a further 30 min, filtered, and left undisturbed in a closed flask at room temperature. X-ray quality, pale green crystals of the product were formed in a period of 2 months. The crystals were collected by filtration, washed with cold MeCN (2 mL) and Et_2_O (2 × 3 mL), and dried in a vacuum desiccator over anhydrous CaCl_2_. The identity of the product was confirmed by unit cell determination. The yield was ∼25–30% (based on the Pr^III^ available). The complex was analyzed satisfactorily as lattice solvent-free, i.e., as **1**. Anal. Calcd for C_44_H_32_Pr_2_N_14_O_16_ (found values in parentheses): C 40.82% (41.03%); H 2.50% (2.41%), and N 15.15% (15.02%). The IR spectrum of the dried powder was identical with that of the authentic sample prepared by method A.

#### Synthesis of [Pr_2_(NO_3_)_4_(L)_2_]·3MeNO_2_ (**2**·3MeNO_2_)

To a pale green slurry of Pr­(NO_3_)_3_·6H_2_O (0.435 g, 1.00 mmol) in MeNO_2_ (10 mL) was added slowly a colorless solution of dpkoxH (0.199 g, 1.00 mmol) and Et_3_N (0.14 mL, 1.00 mmol) in the same solvent (15 mL). The resulting green suspension was stirred, filtered, and the filtrate was left undisturbed for slow evaporation at 5–6 °C. X-ray quality, pale green crystals of the product were precipitated after 2 months. The crystals were collected by filtration, washed with cold MeNO_2_, and dried in air. The yield was 28% (based on the Pr^III^ available). The complex was analyzed satisfactorily as **1**·3MeNO_2_ (i.e., the crystallographic and analytical formulas are the same). Anal. Calcd for C_47_H_41_Pr_2_N_17_O_22_ (found values in parentheses): C 38.19% (38.47%); H 2.80% (2.73%), and N 16.12% (15.88%). Selected IR data (KBr, cm^–1^): 1595­(m), 1563(s), 1468(s), 1432­(m), 1384(s), 1370­(m), 1279(s), 1001(s), 799­(m), 756­(m), 675­(m), 632­(m), 581­(w).

#### Synthesis of [Pr_4_(OH)_2_(NO_3_)_4_(dpkox)_6_(EtOH)_2_]·2MeCN (**3**·2MeCN)

To a pale green solution of Pr­(NO_3_)_3_·6H_2_O (0.435 g, 1.00 mmol) and dpkoxH (0.199 g, 1.00 mmol) in MeCN (10 mL) was added slowly NBu_4_^*n*^OEt/_EtOH_ (0.85 mL, 1.00 mmol). The resulting green solution was stirred for 30 min, filtered, and stored in a flask at room temperature. X-ray quality, pale green crystals of the product were precipitated in a period of 1 week. The crystals were collected by filtration, washed with cold EtOH (2 mL) and Et_2_O (2 × 3 mL), and dried in air. The yield was 55% (based on the Pr^III^ available). The complex was analyzed satisfactorily as lattice MeCN-free, i.e., as **3**. Anal. Calcd for C_70_H_62_Pr_4_N_22_O_22_ (found values in parentheses): C 39.52% (40.00%); H 2.94% (3.01%), and N 14.49% (14.33%). Selected IR data (KBr, cm^–1^): 3390­(mb), 1594(s), 1471(s), 1456(s), 1432(s), 1384(s), 1302(s), 1154­(m), 1083(s), 1028(s), 964­(m), 786­(m), 746­(m), 696(s), 595­(m).

#### Synthesis of [Pr_9_O_4_(OH)_4_(NO_3_)_4_(dpkox)_12_(H_2_O)_4_]·4EtOH·4­(*n*-hexane) (**4**·4EtOH·4­(*n*-hexane))

To a pale green solution of Pr­(NO_3_)_3_·6H_2_O (0.217 g, 0.50 mmol) and dpkoxH (0.199 g, 1.00 mmol) in EtOH (15 mL) was added slowly Et_3_N (0.18 mL, 2.00 mmol). The resulting green solution was stirred for 30 min, filtered, and left undisturbed in a closed flask. After 2 days, *n*-hexane (2 mL) was added to the solution. X-ray quality, green crystals of the product were precipitated after 2 weeks. The crystals were collected by filtration, washed with cold EtOH (2 mL) and Et_2_O (2 × 3 mL), and dried in a vacuum desiccator over silica gel. The yield was 40% (based on the Pr^III^ available). The complex was analyzed satisfactorily as lattice solvents free, i.e., as **4**. Anal. Calcd for C_132_H_108_Pr_9_N_40_O_36_ (found values in parentheses): C 38.68% (38.90%); H 2.66% (2.73%), and N 13.67% (13.37%). Selected IR data (KBr, cm^–1^): 3380­(mb), 1594­(m), 1470(s), 1432(s), 1384(s), 1310(s), 1064­(m), 966­(m), 790­(m), 748­(m), 696­(m), 624­(m).

#### Single-Crystal X-ray Crystallography

Crystals of **1**·3MeCN, **2**·3MeNO_2_, **3**·2MeCN and **4**·4EtOH·4­(*n*-hexane) were taken from the mother liquor and immediately cooled to −113 °C (**1**·3MeCN, **2**·3MeNO_2_ and **3**·2MeCN) or −83 °C (**4**·4MeOH·4­(*n*-hexane)); their dimensions were 0.13 × 0.28 × 0.40 mm^3^, 0.24 × 0.29 × 0.41 mm^3^, 0.07 × 0.14 × 0.32 mm^3^ and 0.32 × 0.34 × 0.54 mm^3^, respectively. Diffraction data were collected on a Rigaku R-AXIS SPIDER Image Plate diffractometer using graphite-monochromated Cu Kα radiation. Data collection (ω-scans) and processing (cell refinement, data reduction and empirical absorption correction) were performed using the CrystalClear program package.^69^ Important crystallographic data are listed in Table S1. The structures were solved by direct methods using SHELXS, ver. 2013/1,^70^ and refined by full-matrix least-squares techniques on *F* ^2^ with SHELXL, ver. 2014/6.^71^ Further experimental crystallographic details for **1**·3MeCN:2θ_max_ = 130°; reflections collected/unique/used, 34145/9139 (*R*_int_ = 0.0825)/9139; 785 parameters refined; (Δ/σ)_max_ = 0.008; (Δρ)_max_/(Δρ*)*_min_ = 1.799/–1.247 e Å^–3^; *R*_1_/*w*R**_2_ (for all data), 0.0719/0.1872. Further experimental crystallographic details for **2**·3MeNO_2_: 2θ_max_ = 130°; reflections collected/unique/used, 46922/9355 (*R*_int_ = 0.0613)/9355; 856 parameters refined; (Δ/σ)_max_ = 0.093; (Δρ)_max_/(Δρ*)*_min_ = 1.943/–1.405 e Å^–3^; *R*_1_/*w*R**_2_ (for all data), 0.0547/0.1500. Further experimental crystallographic details for **3**·2MeCN:2θ_max_ = 130°; reflections collected/unique/used, 32377/6647 (*R*_int_ = 0.0795)/6647; 565 parameters refined; (Δ/σ)_max_ = 0.073; (Δρ)_max_/(Δρ*)*_min_ = 1.717/–1.938 e Å^–3^; *R*_1_/*w*R**_2_ (for all data), 0.0573/0.1396. Further experimental crystallographic details for **4**·4EtOH·4­(*n*-hexane):2θ_max_ = 130°; reflections collected/unique/used, 56010/7732 (*R*_int_ = 0.0660)/7732; 489 parameters refined; (Δ/σ)_max_ = 0.002; (Δρ)_max_/(Δρ*)*_min_ = 1.331/–0.747 e Å^–3^; *R*_1_/*w*R**_2_ (for all data), 0.0486/0.1262. The sites and the thermal parameters of some atoms in **4**·4EtOH·4­(*n*-hexane) were refined using soft SHELXL restraints (DELU, SAME). The presence of lattice solvent molecules in **4**·4EtOH·4­(*n*-hexane) could be easily seen by the residual peaks located in certain areas of the unit cell. Unfortunately, they were disordered so badly and thus they could not be modeled even with restraints. Consequently, SQUEEZE (from PLATON^72^) was used to calculate the void space, the electron count and to get a new hkl file. Based on the electron count derived from the SQUEEZE procedure, the estimated solvent content is 4EtOH and 4­(*n*-hexane) per cluster molecule. The H atoms were either located by different maps and were refined isotropically or were introduced at calculated positions as riding on their corresponding bonded atoms. All non-H atoms were refined anisotropically. Plots of the structures were drawn using the Diamond program package.^73^

## Results and Discussion

### Synthetic Comments

The principal goal of this work was to explore the to-date unknown 4f-metal chemistry of dpkoxH, targeting at the isolation of new complexes with novel structural motifs and potentially interesting properties. For reasons explained in “Introduction section”, we started our efforts with Pr­(III). The 1:1 reaction between Pr­(NO_3_)_3_·6H_2_O and dpkoxH in MeCN gave a green solution, from which pale green crystals of [Pr_2_(NO_3_)_4_(L)_2_]·3MeCN (**1**·3MeCN) were subsequently isolated in a low yield (∼20%). Somewhat to our surprise, the dpkoxH or/and dpkox^–^ ligands had not been incorporated in the product; the incorporated organic ligand was the monoanion of di­(pyridin-2-yl)­methanone *O*-(1-hydroxy-1,1-di­(pyridin-2-yl))­methyl oxime (for the structural formula of the neutral molecule (LH), see Chart 2). The ligand L^–^ was unknown in inorganic chemistry and the neutral molecule (LH) has never been synthesized in organic chemistry. The same product was obtained by adding Et_3_N in the reaction solution, i.e., from the 1:1:1 Pr­(NO_3_)_3_·6H_2_O/dpkoxH/Et_3_N system; the yield was slightly higher (25–30%), but still low. In order to investigate if the solvent plays a role in the dpkoxH → L^–^ transformation, we replaced MeCN with MeNO_2_ in an otherwise similar reaction system. The isolated product was [Pr_2_(NO_3_)_4_(L)_2_]·3MeNO_2_ (**2**·3MeNO_2_), either in the presence of Et_3_N (this procedure is described in the “Experimental section”) or without addition of this base. Again the yield was slightly higher (∼30%) in the presence of the base compared to that from the Et_3_N-free system (∼20%). In all cases, the crystallization process was very slow ranging from ca. 1 week to 2 months. The transformation is metal ion-assisted/promoted as proven by “blind” experiments using exactly the same concentrations. In the first set of experiments, a solution of dpkoxH in MeCN or MeNO_2_, containing 1 or 2 drops of water to account for the H_2_O present in Pr­(NO_3_)_3_·6H_2_O, was slowly evaporated at room temperature until dryness. The IR spectra of the well dried residues were identical with the spectrum of dpkoxH. The same experiments were repeated in the presence of an equimolar amount of Et_3_N, and the obtained residues were perfectly analyzed as (Et_3_NH)­(dpkox).

Vanadium- and manganese-assisted/promoted reactivity of dpkoxH has been reported in the past (Scheme 1). The reactions of [V^III^Cl_3_(THF)_3_] and dpkoxH provided access to the vanadyl complexes [V^IV^OCl_2_(dpi)­(THF)], [V^IV^OCl_2_(adpm)] and [V^IV^OCl_2_(adpe)].^19^ Reactions of Mn^II^ and Mn^VII^ sources with dpkoxH in the presence of simple carboxylate ions (RCO_2_^–^) led to the mixed-valence coordination clusters [Mn_3_^II^Mn_3_^III^O_2_(O_2_CR)_6_(dpkox)_2_(dpk·OH)_2_]­(ClO_4_)^26^ and [Mn_2_^II^Mn_2_^III^(O_2_CR)_2_X_2_(dpkox)_2_(dpk·O)_2_];^74^ the anionic ligand X^–^ is Cl^–^, Br^–^ and NO_3_^–^, while dpk·OH^–^ and dpk·O^2–^ are the monoanions and dianions, respectively, of the *gem*-diol derivative (*vide infra*) of di-2-pyridyl ketone (dpk, Chart 3). The observed dpkoxH → L^–^ transformation is unprecedented.

**Scheme 1:**
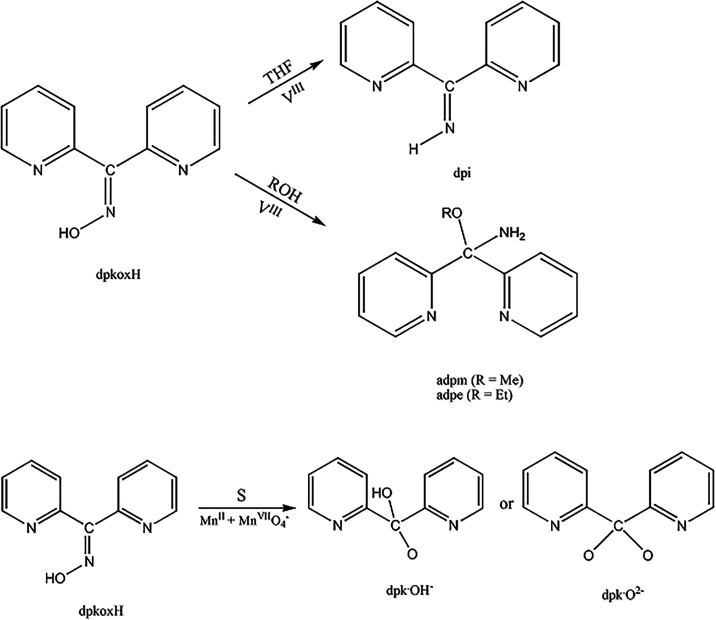
Previously Reported Metal Ion-Assisted/Promoted Transformations of dpkoxH^a^

a The molecules dpi, adpm, adpe, and the anions dpk·OH^–^, dpk·O^2–^ are incorporated as ligands in the metal complexes. S = MeCN, EtOH, CH_2_Cl_2_, or a combination of them.

**Chart 3:**
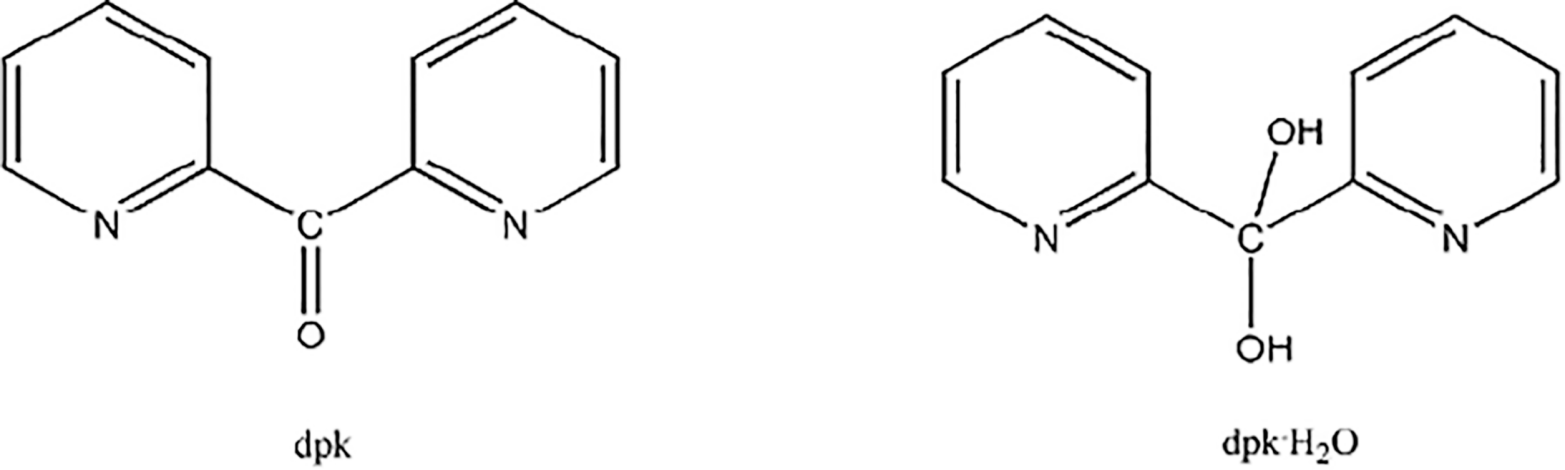
Structural Formulae of Di-2-pyridyl Ketone (dpk) and its Neutral *Gem*-Diol Form (dpk·H_2_O)[Fn c3fn1]

a The *gem*-diol form dpk·H_2_O and its anionic species (dpk·OH^–^, dpk·O^2–^; Scheme 1) do not exist free, but only in the presence of metal ions, i.e., as ligands in complexes.

The *stoichiometric* reaction of Pr­(NO_3_)_3_·6H_2_O and dpkoxH in the absence or in the presence of Et_3_N in MeCN that leads to complex [Pr_2_(NO_3_)_4_(L)_2_]·3MeCN (**1**·3MeCN) is depicted in Scheme 2. Four equivs of dpkoxH are required for two equivs of Pr­(NO_3_)_3_·6H_2_O to produce one equiv of the complex and two equivs of free hydroxylamine (H_2_NOH) in the presence of Et_3_N or two equivs of hydroxylammonium nitrate in the absence of the base. Obviously, two molecules of dpkoxH are hydrolyzed during the course of the reaction to the corresponding di-2-pyridyl ketone, dpk, and the other two are added to the dpk molecules produced giving rise to two L^–^ ions.

**Scheme 2:**
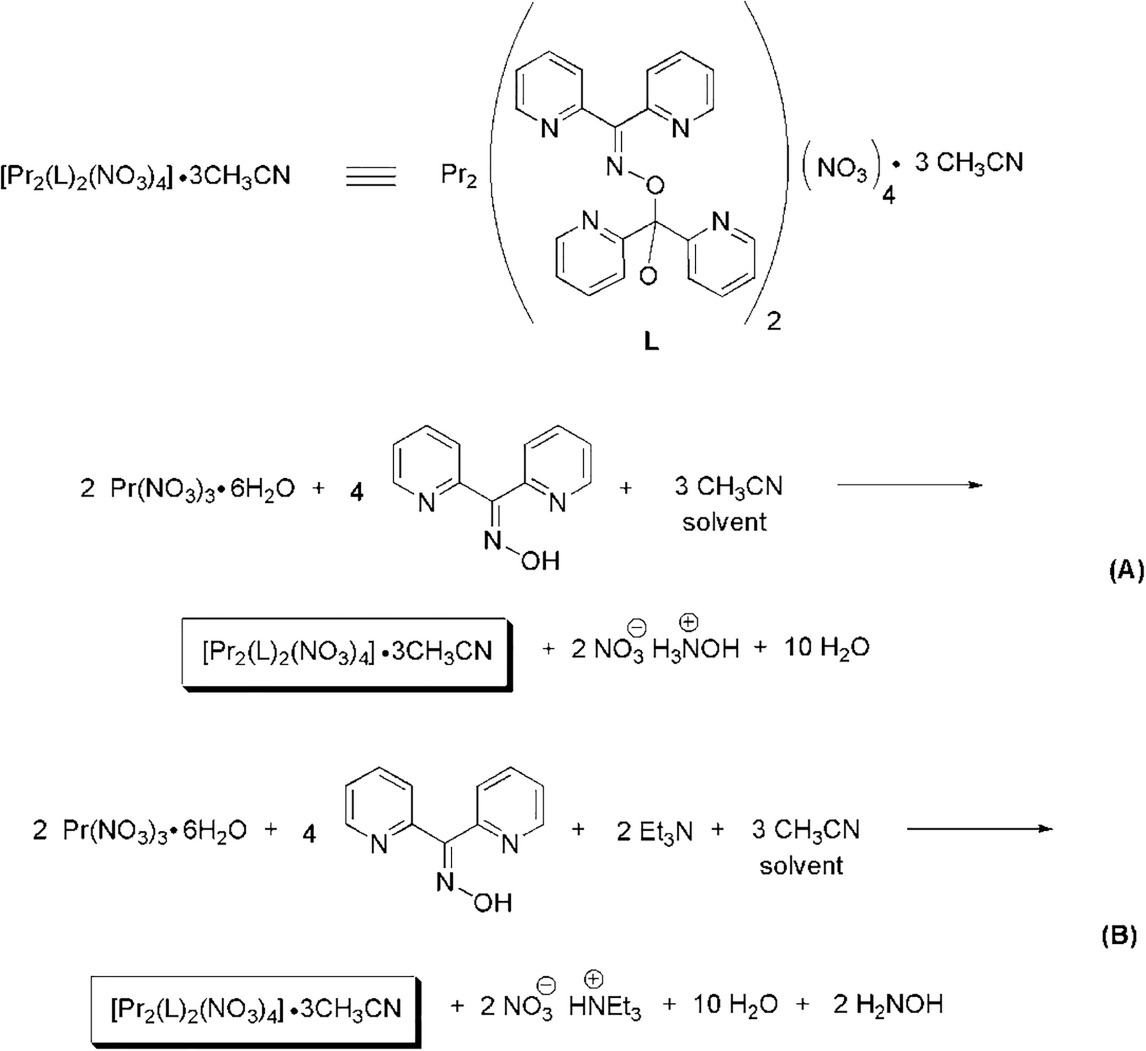
Equations That Represent the Stoichiometric Reactions of Pr­(NO_3_)_3_·6H_2_O with dpkoxH in the Absence (A) and the Presence (B) of Et_3_N

In order to increase the yields (∼25%) and shorten the crystallization time (period of months) for the preparation of the two dinuclear Pr­(III)/L^–^ complexes, we used a stronger base than Et_3_N. These goals were achieved, but the identity of the product changed! Replacing Et_3_N (p*K*_a_ = 10.7) with NBu_4_^*n*^OEt (p*K*_a_ EtOH = 15.9), and keeping all the synthetic and crystallization parameters constant, compared to those used for the preparation of **1**·3MeCN (Method B), the tetranuclear cluster [Pr_4_(OH)_2_(NO_3_)_4_(dpkoxH)_6_(EtOH)_2_]·2MeCN (**3**·2MeCN) was isolated; the yield was ∼55% and the crystallization process rather fast (∼1 week). Presumably, the stronger base does not favor the dpkoxH → L^–^ transformation producing large concentrations of hydroxides which are incorporated in the complex. The strong base also changes the kinetics of the crystallization process, again disfavoring the transformation whose crystallization appears slow. The stoichiometric reaction that leads to **3** is represented by eq 1.

1 $$4\mathrm{Pr}{\left({\mathrm{NO}}_{3}\right)}_{3}\cdot 6H_{2}O+6\mathrm{dpkoxH}+8{\mathrm{NBu}}_{4}^{n}\mathrm{OEt}\overset{\mathrm{MeCN}}{\to }\left[{\mathrm{Pr}}_{4}{\left(\mathrm{OH}\right)}_{2}{\left({\mathrm{NO}}_{3}\right)}_{4}{\left(\mathrm{dpkox}\right)}_{6}{\left(\mathrm{EtOH}\right)}_{2}\right]+8{\mathrm{NBu}}_{4}^{n}\left({\mathrm{NO}}_{3}\right)+6\mathrm{EtOH}+22H_{2}O$$

Complexes **1**–**3** were prepared in an aprotic solvent (MeCN, MeNO_2_) using a Pr­(III)/dpkoxH/base molar ratio of 1:1:1. We thought that reactions in protic solvents employing different molar ratios could give a different chemistry. In particular, we sought to investigation reactions with use of an excess of base hoping that large concentrations of OH^–^s (“hard” base according to the HSAB model) would create an environment for stabilization of Pr­(IV), which is a very “hard” acid [“harder” than Pr­(III)] according to the same model; we also hoped that precipitation of amorphous hydroxide/oxide species would be avoided, because of the presence of dpkoxH which could help in the isolation of molecular complexes. Our goal was proven to be both unsuccessful and successful. Unsuccessful because we failed to prepare an all-Pr­(IV) complex, but successful because a mixed-valence Pr­(III/IV) cluster was isolated. After many efforts, we arrived at the optimized procedure described in “Experimental Section”. The Pr­(NO_3_)_3_·6H_2_O/dpkoxH/Et_3_N (1:2:4) reaction mixture in EtOH led to a green solution, from which were subsequently isolated pale green crystals of [Pr_8_^III^Pr^IV^O_4_(OH)_4_(NO_3_)_4_(dpkox)_12_(H_2_O)_4_]·4EtOH·4­(*n*-hexane) [**4**·4EtOH·4­(*n*-hexane)] in moderate yield. The stoichiometric reaction is represented by eq 2. The same product (analytical and IR evidence) was obtained by employing NBu_4_^*n*^OEt, under otherwise identical synthetic and crystallization conditions, in a comparable (or slightly higher) yield. Further increase of the equivalents of bases (B), i.e., Pr­(NO_3_)_3_·6H_2_O/dpkoxH/B (1:2:6), led to amorphous products which could not be characterized further. Due to solubility problems in suitable solvents (e.g., THF), cyclic voltammetry studies could not be performed.

2 $$9{\mathrm{Pr}}^{\mathrm{III}}{\left({\mathrm{NO}}_{3}\right)}_{3}\cdot 6H_{2}O+12\mathrm{dpkoxH}+23{\mathrm{Et}}_{3}N+\frac{1}{4}O_{2}\overset{\mathrm{EtOH}}{\to }\left[{\mathrm{Pr}}_{8}^{\mathrm{III}}{\mathrm{Pr}}^{\mathrm{IV}}O_{4}{\left(\mathrm{OH}\right)}_{4}{\left({\mathrm{NO}}_{3}\right)}_{4}{\left(\mathrm{dpkox}\right)}_{12}{\left(H_{2}O\right)}_{4}\right]+23\left({\mathrm{Et}}_{3}\mathrm{NH}\right)\left({\mathrm{NO}}_{3}\right)+42.5H_{2}O$$

### IR Spectra in Brief

In the IR spectra of the well-dried and analytically pure samples **3** and **4**, the medium intensity broad band at ∼3385 cm^–1^ is assigned to the ν­(OH) vibration of the coordinated EtOH (**3**) and H_2_O (**4**) molecules, combined with the ν­(OH)_hydroxido_ mode. The bands at 1563 and 1375 cm^–1^ in the spectrum of **2**·3MeNO_2_ are attributed to the ν_as_(NO_2_) and ν_s_(NO_2_) vibrations, respectively, of the −NO_2_ nitromethane group, the former overlapping with a pyridyl stretching vibration.^75,76^ The latter band is absent from the spectra of the other compounds, as expected. A strong band at 1384 cm^–1^ is common in the spectra of the four compounds. This band can be safely assigned^77^ to the ν_3_(*E*′)­[ν*_d_* (NO)] vibrational mode of the ionic planar nitrates (*D*_3*h*_); such nitrates do not exist in the crystal structures of the complexes. The appearance of this mode is due to the replacement (partial or complete) of the bidentate nitrato ligands by bromides that are present in excess in the KBr matrix used for the preparation of the pellets, and the production of KNO_3_ (containing ionic nitrates); this replacement is facilitated by the applied pressure.^78,79^ This explanation is further confirmed by the absence of the ∼1385 cm^–1^ band from the mull IR spectra of the complexes in hexachlorobutadiene. The solid-state reaction is represented by eq 3 for complexes **1** and **2** (*x* ≥ 1).

3 $$\left[{\mathrm{Pr}}_{2}{\left({\mathrm{NO}}_{3}\right)}_{4}{\left(L\right)}_{2}\right]+\mathrm{x KBr}\overset{\mathrm{KBr}}{\underset{P}{⇄}}\left[{\mathrm{Pr}}_{2}{\left({\mathrm{NO}}_{3}\right)}_{4-x}{\mathrm{Br}}_{x}{\left(L\right)}_{2}\right]+{\mathrm{x KNO}}_{3}$$

### Description of Structures

The crystal structures of the four complexes were determined by single-crystal X-ray crystallography. Structural plots are presented in Figures 1–5 and S1–S21. The coordination modes of L^–^ and dpkox^–^ in the compounds are shown in Chart 4, while the core of **3** is presented in Chart 5. Bond lengths and angles can be found in the corresponding.cif files.

**Figure 1:**
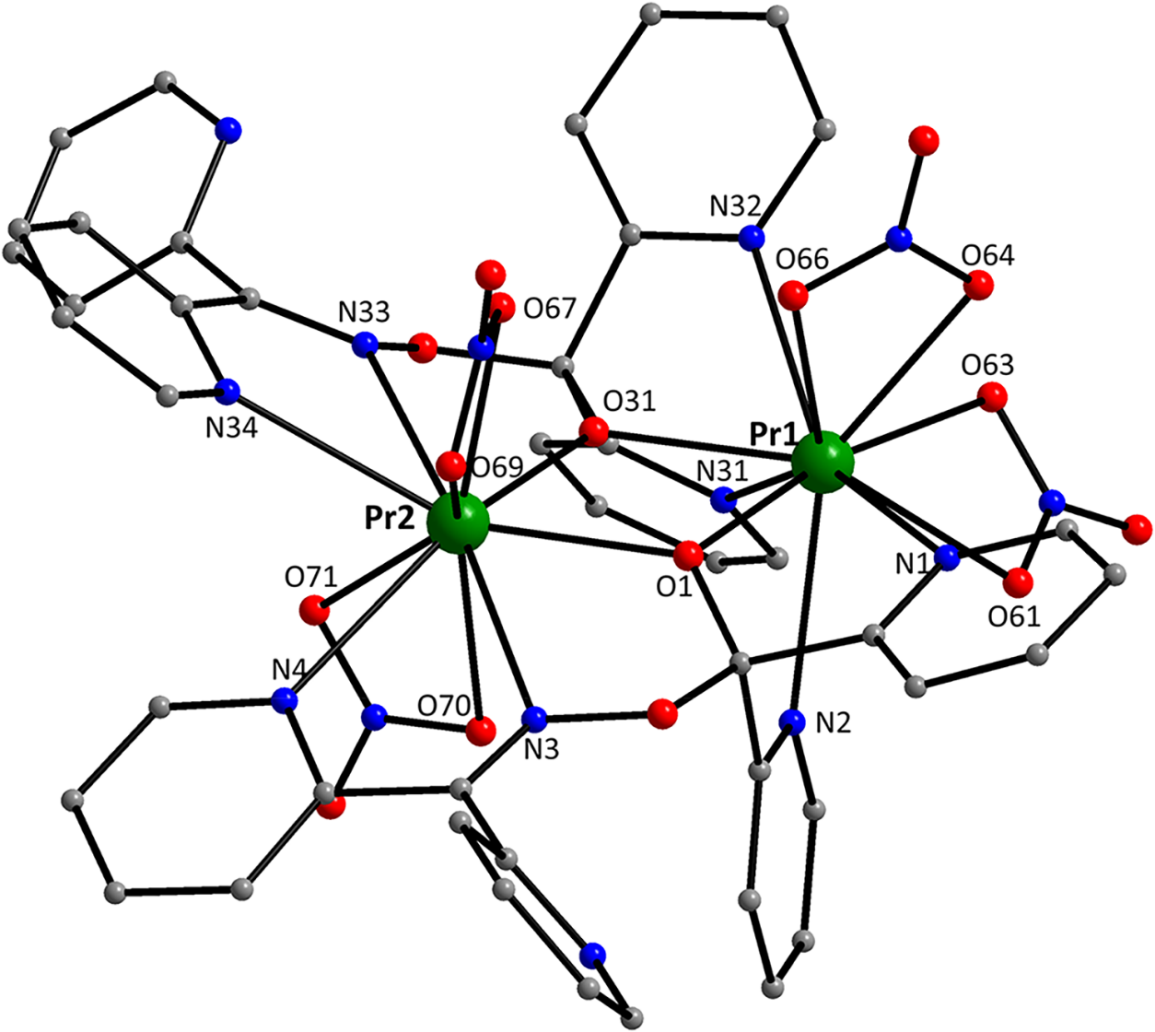
Structure of the molecule [Pr_2_(NO_3_)_4_(L)_2_] that is present in the crystal of **2**·3MeNO_2_. Diagnostic interatomic distances (Å): Pr–O_alkoxido_ = 2.392(3)–2.419(4), Pr–O_nitrato_ = 2.543(4) 2.654(4), Pr–N_pyridyl_ = 2.653(5)–2.780(4), Pr2–N3 = 2.670(5), Pr2–N33 = 2.703(4), C17–N3 = 1.285(7), N3–O2 = 1.401(6), C47–N33 = 1.288(7), N33–O32 = 1.388(5), Pr1···Pr2 = 4.044(1). Selected bond angles (°): O64–Pr1–O66 = 49.2(1), O70–Pr2–O71 = 50.0(1), O1–Pr1–O63 = 170.2(1), N3–Pr2–N33 = 173.0(1), Pr1–O1–Pr2 = 113.2(1), Pr1–O31–Pr2 = 115.0(1).

**Chart 4:**
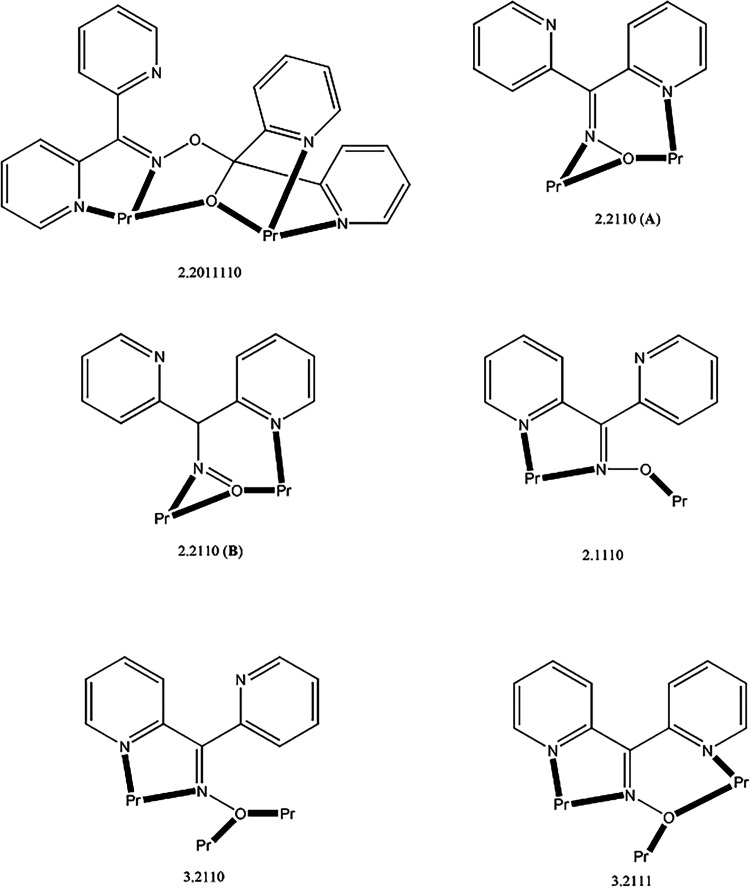
Coordination Modes of the Ligands L^–^ and dpkox^–^ in the Structures of the Four Complexes Presented in This Work and the Harris Notation That Describes These Modes[Fn c4fn1],[Fn c4fn2]

a The 2.2110­(**A**) and 2.2110­(**B**) modes are simplified representations of the real ligation modes (*vide infra*).
b The coordination bonds are drawn with bold lines.

**Chart 5:**
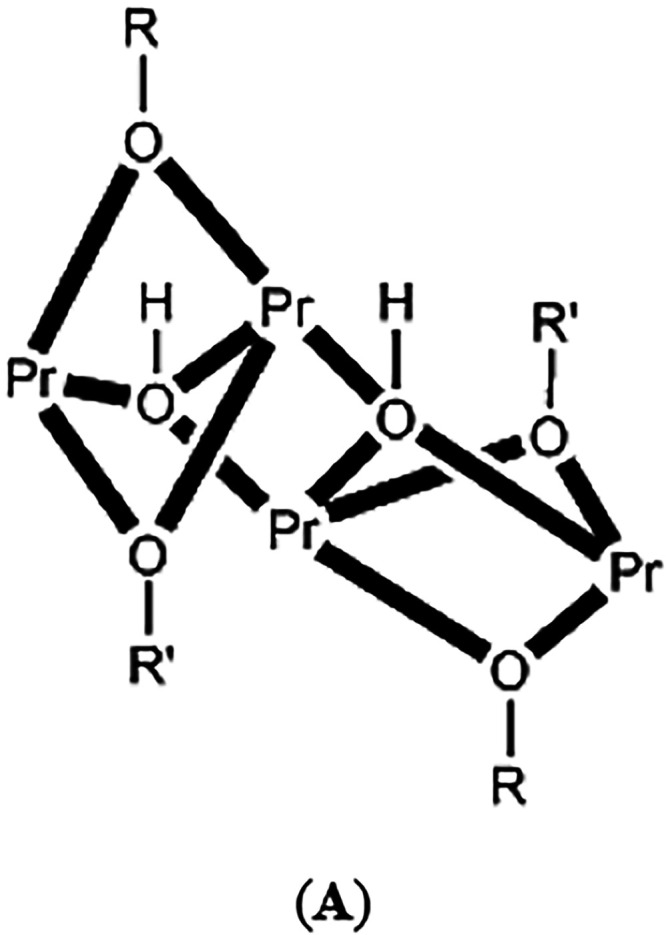
{Pr_4_(μ_3_–OH)_2_(μ_2_-OR)_2_(μ_2_-OR′)_2_}^6+^ Core of 3[Fn c5fn1]

a RO^–^ is the 2.2110 (**A**) ligand and R’O^–^ the 3.2110 one (see Chart 4). The coordination bonds are drawn with bold lines.

The crystal structures of **1**·3MeCN (Figures S5–S11) and **2**·3MeNO_2_ (Figures 1, 2 and S1–S4) consist of dinuclear [Pr_2_(NO_3_)_4_(L)_2_] and lattice solvent molecules. Since the dinuclear molecules have strikingly similar structures, only the molecular structure of **2** will be discussed. The two Pr^III^ centers are bridged by the deprotonated oxygen atoms (O1, O31) of two “head-to-head” η^1^:η^1^:η^2^:η^1^:η^1^:μ_2_ (or 2.2011110 using Harris notation;^80^ Chart 4) L^–^ ligands, with the Pr1···Pr2 distance being 4.044(1) Å. The Harris Notation method describes the coordination mode of a ligand as X.Y_1_Y_2_Y_3_···Y*_n_*. *X* is the number of metal ions bound by the donor atoms of the ligand, and each *Y* value refers to the number of metal sites attached to the different donor atoms. The order of the *Y* groups follows the Cahn-Ingold-Prelog priority rules, and hence O is placed before N in the present work. When one (or more) donor atom, e.g., N is coordinated, whereas another (or other) donor atom of a similar nature, e.g., N is not coordinated, an integer number (1, 2, ···) is assigned to the coordinated donor atom(s) in decreasing order, and then nil(0) is used for the “free” one(s). Four pyridyl nitrogen atoms (N1, N2, N31, N32) whose rings are connected to the carbons with the single C–O bonds (C6, C36) and two bidentate chelating nitrato groups complete 10-coordination at Pr1. Pr2 is also 10-coordinate. Its coordination sphere is completed by two “oximato-type” nitrogen (N3, N33) and two pyridyl nitrogen atoms (N4, N34) from two different L^–^ ligands, as well as by two chelating nitrato groups; the coordinated 2-pyridyl rings are connected to the “oximato-type” carbon atoms (C17, C47). Thus, the coordination spheres of the two metal centers are {Pr1­(O_nitrato_)_4_(O_alkoxido_)_2_(N_pyridyl_)_4_} and {Pr2­(O_nitrato_)_4_(O_alkoxido_)_2_(N_pyridyl_)_2_(N_oximato_)_2_}. The Pr^III^–O-Pr^III^ bridges are nearly symmetrical. The C6–O1, C6–O2, C36–O31 and C36–O32 bond lengths are in the range 1.350(6)–1.469(7) Å, indicating essentially single carbon–oxygen bonds. The bond angles around C6 and C36 are in the ranges 104.7(4)–112.2(4)° and 106.0(4)–113.0(4)°, respectively, confirming the sp^3^ character of these carbon atoms. The C17–N3 and C47–N33 distances are 1.285(7) and 1.288(7) Å, respectively, typical of “oximato-type” double carbon–nitrogen bonds. The sp^2^ character of these carbon bonds is further supported by the C–C–C and N–C–C bond angles which are in the range 114.6(5)–125.1(5)°.

**Figure 2:**
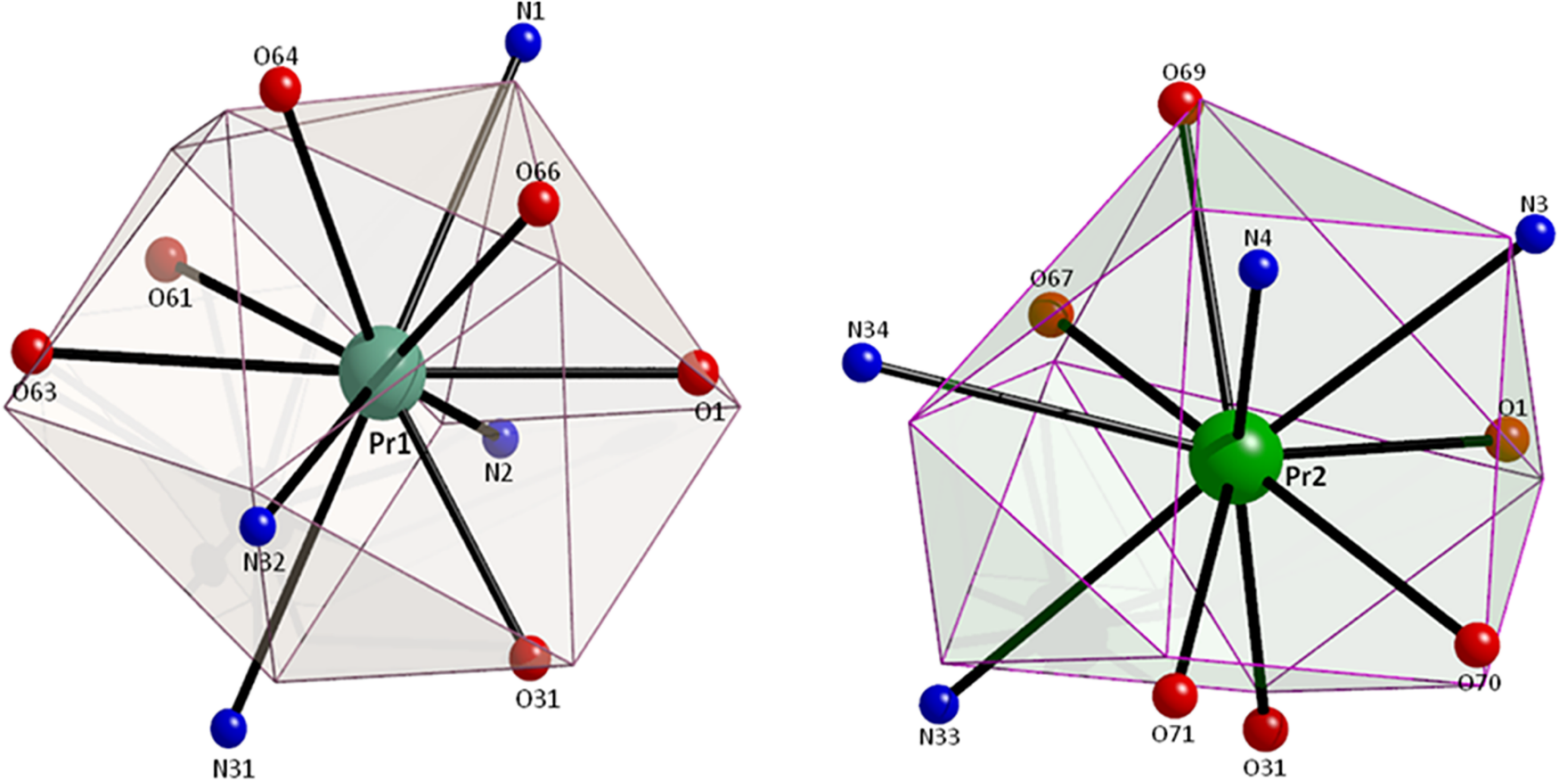
Coordination polyhedra of Pr1 and Pr2 in the structure of **2**·3MeNO_2_. The very small spheres represent the vertices of the ideal polyhedra.

Using the program SHAPE,^81^ the coordination polyhedra of Pr1 and Pr2 can be described as distorted sphenocorona (CshM = 4.016) and distorted tetradecahedron (CshM = 3.746), respectively (Figure 2).

There are two weak H-bonding interactions within the dinuclear molecule, with aromatic carbons as donors and the noncoordinated 2-pyridyl nitrogens (N5, N35) as acceptors (Figure S1). The molecules form layers parallel to the (001) planes through C–H···O interactions (Figure S3), while molecules belonging to neighboring layers and stacked along the *c* axis interact further (C–H···O and π–π stacking interactions) creating the 3D architecture of the structure (Figure S4). Details for the supramolecular structural features of **1**·3MeCN and **2**·3MeNO_2_ are provided in the Electronic Supporting Information. It should be noted that the two compounds have almost identical cell dimensions and crystallize in the same space group. However, their crystal packing is different due to the different lattice solvent molecules included in the structures.

The crystal structure of **3**·2MeCN (Figures 3 and S12–S17) consists of tetranuclear [Pr_4_(OH)_2_(NO_3_)_4_(dpkox)_6_(EtOH)_2_] cluster molecules and lattice MeCN solvents. There is a crystallographically imposed inversion center at the midpoint of the Pr1···Pr1′ (or Pr2···Pr2′) vector. The tetranuclear molecule is held together by two symmetrical μ_3_ (or 3.3) hydroxido groups, two 2.2110­(**A**), two 2.1110 and two 3.2110 dpkox^–^ ligands (Chart 3). Peripheral ligation is completed by four chelating nitrato groups at Pr2/2′, while a terminal EtOH molecule is coordinated to Pr1 (and its symmetry equivalent). Thus, the core is {Pr_4_(μ_3_–OH)_2_(μ_2_-O_oximato_)_4_}^6+^ (Chart 5). The {Pr_4_(μ_3_–OH)_2_}^10+^ unit of the core comprises four strictly (by symmetry) coplanar Pr^III^ ions in a “butterfly”-type disposition and two triply bridging (μ_3_) hydroxido (O61, O61′) groups. Pr1 and Pr1′ occupy the “body” sites, and Pr2 and Pr2′ occupy the “wingtip” sites. The two μ_3_–OH^–^ ions are above and below the Pr_4_ plane (ca. 0.90 Å). This is also reflected in the sums of the Pr–O–Pr angles around the hydroxido groups with deviate from 360° by ca. 40°, and are close to the ideal value of 328.4° expected for sp^3^ hybridization. The two “body” Pr^III^ ions are bridged by two μ_3_–OH^–^ groups, while a single μ_3_–OH^–^ also bridges a “wingtip” metal ion. The two 3.2110 dpkox^–^ ligands are coordinated to the “body” Pr1 and Pr1′ centers through one pyridyl N, the oximato N and the oximato O atoms, and they use the latter to bridge one of the “wingtip” Pr2 and Pr2′ ions. The two 2.1110 dpkox^–^ ligands each chelates one “wingtip” metal ion through its pyridyl and oximato N atoms, while its oximato O atom is coordinated to one “body” metal ion. Each 2.2110­(**A**) dpkox^–^ ligand connects one “body” and one “wingtip” Pr^III^ ions. The coordination to the former involves an unusual three-membered “chelating” ring with the oximato N and O atoms being the donor sites, while the oximato O and one pyridyl N atoms are bonded to the latter; in this way the oximato oxygens O11 and O11′ are doubly bridging. The Pr–O–Pr bridges through O11/O11′ are exactly symmetrical (*vide infra*), whereas those involving the oxygens of the 3.2110 ligands are asymmetrical [Pr1/1′-O1/1′ = 2.638(4) Å, Pr2/2′-O1′/1 = 2.401(4) Å]. The “body” metal ions are 9-coordinate and the “wingtip” ones are 10-coordinate; the coordination spheres are {Pr1/1′(O_hydroxido_)_2_(O_oximato_)_3_(O_ethanol_)­(N_pyridyl_)_2_(N_oximato_)} and {Pr2/2′(O_hydroxido_)­(O_nitrato_)_4_(O_oximato_)_2_(N_pyridyl_)­(N_oximato_)_2_}. The coordination polyhedra of Pr1/1′ and Pr2/2′ can be described as muffin (CShM = 1.070) and distorted sphenocorona (CShM = 4.301), respectively (Figure S13).

**Figure 3:**
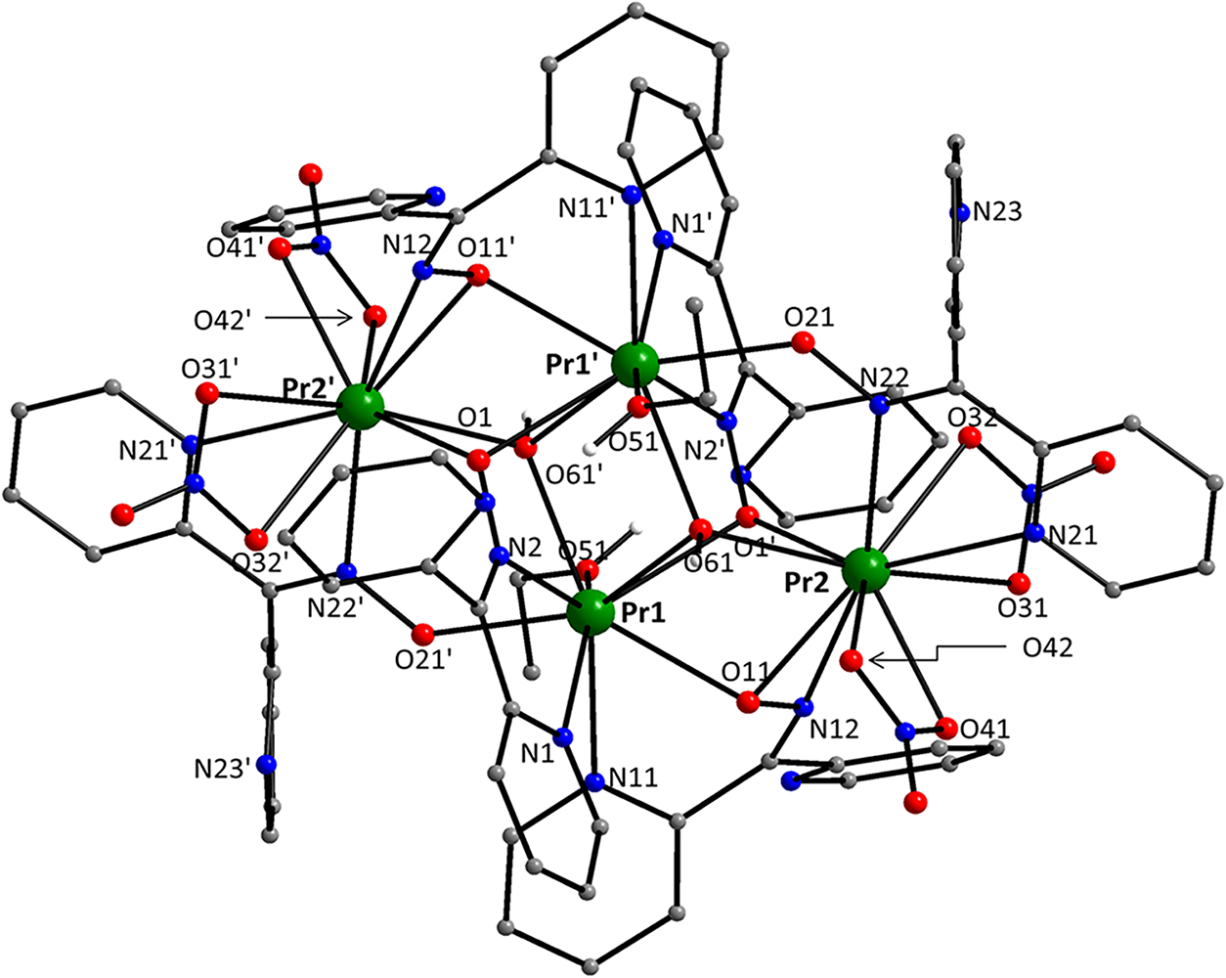
Structure of the molecule [Pr_4_(OH)_2_(NO_3_)_4_(dpkox)_6_(EtOH)_2_] that is present in the crystal of **3**·2MeCN. Diagnostic interatomic distances (Å): Pr–O_hydroxido_ = 2.456(4)–2.506(4), Pr–O_nitrato_ = 2.551(5)–2.611(5), Pr–O_oximato_ = 2.344(4)–2.638(4), Pr–N_pyridyl_ = 2.662(5)–2.753(4), Pr–N_oximato_ = 2.644(5)–2.670(5), Pr1–O51_EtOH_ = 2.541(5), Pr1···Pr1′ = 3.800(4), Pr2···Pr2′ = 7.140(4), Pr1–Pr2 = 3.808(3), Pr1···Pr2′ = 4.268(4), C26–N12 = 1.309(8), N12–O11 = 1.365(6), C6–N2 = 1.294(7), N2–O1 = 1.364(6), C46–N22 = 1.314(8), N22–O21 = 1.344(7). Selected bond angles (°): Pr1–O61–Pr2 = 100.2(2), Pr1–O61′-Pr2′ = 120.5(2), Pr1–O61–Pr1′ = 100.0(1), O11–Pr2–N12 = 30.8(1), Pr2–O11–N12 = 81.9(3), Pr2–N12–O11 = 67.3(2), O31–Pr2–O32 = 49.2(2), N11–Pr1–O61′ = 143.1(1), N12–Pr2–N22 = 162.3(2). Symmetry operation: ′2–*x*, 1–*y*, 1–*z*.

There are two (four by symmetry) intramolecular H bonds (Figure S12). In the first one (which is of moderate strength) the oxygen of the coordinated EtOH ligand (O51/51′) is the donor and the noncoordinated pyridyl nitrogen (N3′/3) of the 3.2110 dpkox^–^ ligand is the acceptor; the dimensions of this classical H bond are O51···N3′ = 2.728(7) Å, H­(O51)···N3′ = 1.80(12) Å and O51–H­(O51)···N3′ 164(11)°. The second type is rather a nonclassical H-bonding interaction with a pyridyl C atom as donor and the coordinated oximato oxygen (O21) of the 2.1110 dpkox^–^ ligand as acceptor.

A notable feature of the molecular structure of **3**·2MeCN is the 2.2110­(**A**) mode (Chart 4) of two crystallographically identical dpkox^–^ ligands, which results in the η^1^:η^2^:μ_2_ coordination of the oximato group; this exhibits a side-on donating NO group whose oxygen is bridging. This *bridging* mode is extremely rare in transition-metal chemistry^82^ and it has been observed only once in 4f-metal chemistry;^83^ the only previously reported Ln­(III) complex that exhibits this ligation is [(cp)_4_Gd_2_(ONCMe_2_)_2_].^83^ The Pr2/2′-N12/12′ [2.664(5) Å], Pr1/1′-O11/11′ [2.463(4) Å] and Pr2/2′-O11/11′ [2.464(4) Å] distances indicate clearly coordination bonds. The Pr1/1′-O11/11′-Pr2/2′ angle is 101.2(1)°. The O11/11′-N12/12′-C26/C26′ angle is 117.8(5)°, typical^84^ for η^1^:η^1^ chelating oximato groups. The Pr1–O11–N12 angle in **3**·2MeCN is 113.4(3)°, whereas the corresponding angles in the organometallic Gd­(III) complex^83^ are near 180°, thus making this grouping unique in the former. The C–N and O–N oximato bond lengths in the six dpkox^–^ ligands are consistent with the description of these bonds as slightly delocalized, i.e., as 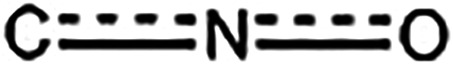. Thus, the nitrogen–oxygen bond distances [1.344(7)–1.365(6) Å] are somewhat shorter than the single-bond N–O distance of 1.392(2) Å observed in the crystal structure of free dpkoxH.^85^ The carbon–nitrogen bond distances [1.294(7)–1.314(8) Å] are slightly longer than the double-bond C=N distance of 1.282(2) Å in the structure of the free ligand.^85^ Despite the delocalized character of all the oximato groups in the present structure, only two dpkox^–^ ligands form the η^1^:η^2^:μ_2_ Pr–N–O-Pr units. The Pr2/2′-N2′/2 and Pr1/1′-N22′/N22 distances [3.160(4) and 3.371(4) Å, respectively] are too long, precluding the η^1^:η^2^:μ_2_ description of the oximato groups for the four other dpkox^–^ ligands in **3**·2MeCN. The terminal (i.e., *chelating*) three-membered 
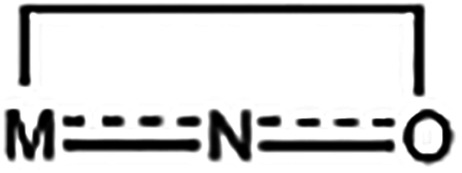
 ring is well-known in transition-,^86^ main-^87^ and actinoid-metal^88–90^ oximato chemistry, but it has not been observed in Ln­(III) chemistry. However, a similar three-membered ring has been observed in 4f-metal complexes, but the ligation mode is well described as η^2^-nitroso, i.e., as 
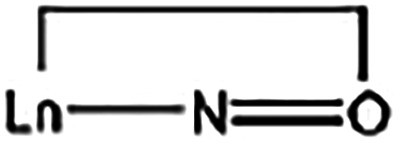
,^91–95^ and not as oximato-type.

Details of the supramolecular structural features of **3**·2MeCN can be found in the Electronic Supporting Information.

Complex **4**·4EtOH·4­(*n*-hexane) crystallizes in the tetragonal space group *I*4_1_/*a* and the structure is described using origin choice 2. Pr1 occupies the 4*a* which is characterized by a 4-fold roto-inversion axis symmetry (−4) and this is the symmetry of the whole molecule. Thus, there are three crystallographically independent metal ions (Pr1, Pr2, Pr3) and three dpkox^–^ ligands. Aspects of the molecular structure of **4** are shown in Figures 4, 5 and S18–S20. Charge-balance considerations, EPR spectroscopy (*vide infra*) and the tetragonal space group suggest that the crystallographically unique Pr1 should be Pr^IV^, thus making the molecule mixed-valence ({Pr_8_^III^Pr^IV^}). The crystal structure also contains lattice EtOH and *n*-hexane solvent molecules. The nine metal ions are held together by four μ_3_-O^2–^ (O1X and symmetry equivalents), four μ_3_–OH^–^ (O2X and symmetry equivalents) groups, as well as four 2.1110 (those containing the oximato nitrogen N22 and symmetry equivalents), four 3.2111 (those containing the oximato nitrogen N2 and symmetry equivalents) and four 2.2110 (**B**) [those containing the oximato nitrogen N42 and symmetry equivalents] dpkox^–^ ligands (Chart 4). Peripheral ligation is completed by four bidentate chelating nitrato groups at Pr3/3′/3″/3‴ and four aqua ligands at the same metal ions. Thus, the core is {Pr_8_^III^Pr^IV^(μ_3_-O)_4_(μ_3_–OH)_4_(μ_2_-OR)_4_(μ_2_-OR′)_4_}^8+^, where RO^–^ is the 2.2110­(**B**) dpkox^–^ ligand and R’O^–^ the 3.2111 one. The metal topology can be described as a triangular dodecahedral arrangement of the eight Pr^III^ ions centered on the tetravalent Pr1 ion (Figure 5, left). The purely inorganic {Pr_8_^III^Pr^IV^(μ_3_-O)_4_(μ_3_–OH)_4_}^16+^ unit of the core (Figure 5, right) can be described as four fused “butterflies”, the Pr^IV^ ion being the common site at one of their “body” positions. This unit is unique in molecular lanthanoid­(III) chemistry. Each “butterfly” possesses one μ_3_-O^2–^ and one μ_3_–OH^–^ groups. The Pr···Pr distances are in the range 3.963(1)-7.889(1) Å; the longest one is Pr2″···Pr2‴ distance, see Figure 4.

**Figure 4:**
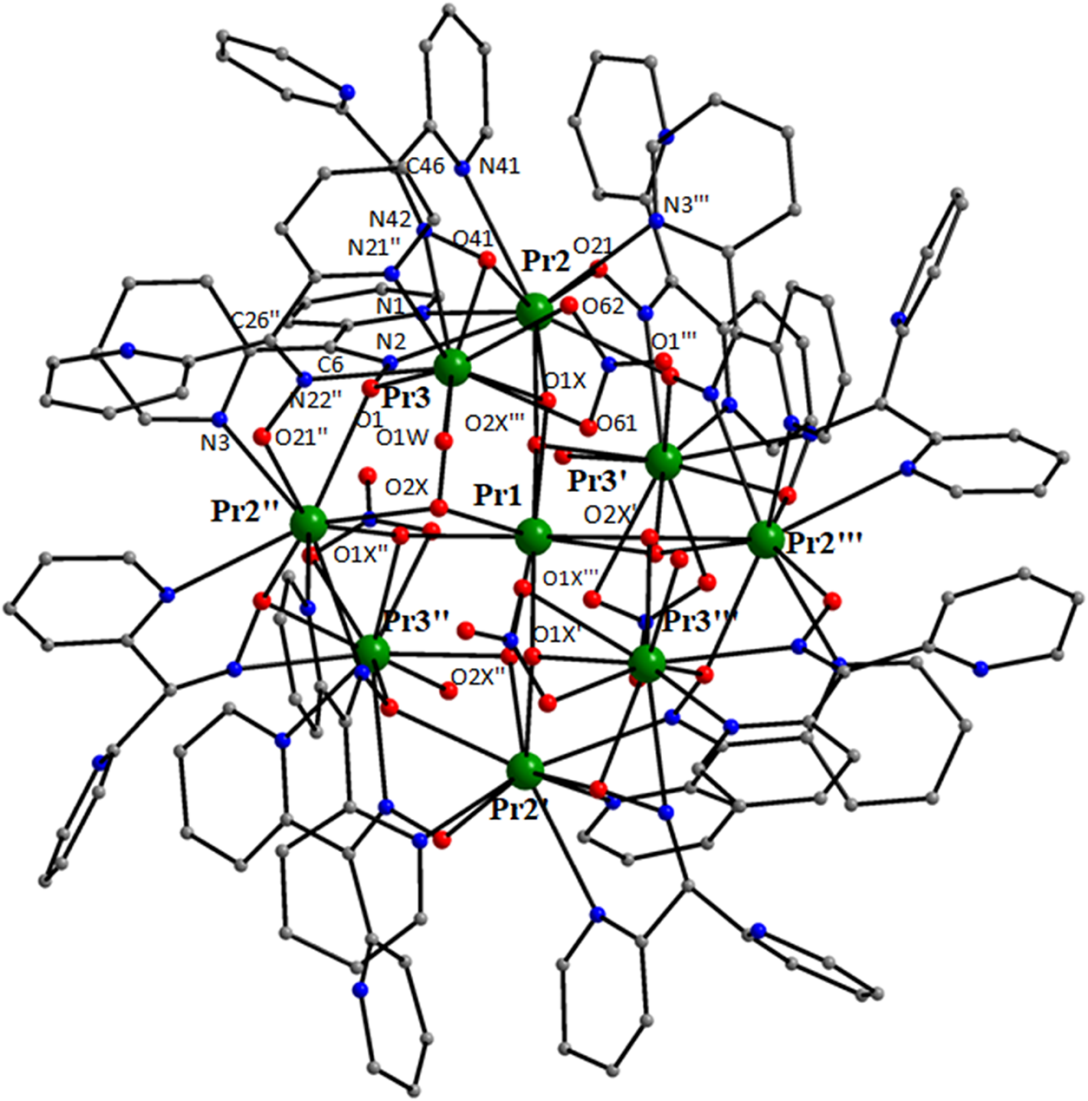
Structure of the molecule [Pr_8_^III^Pr^IV^O_4_(OH)_4_(NO_3_)_4_(dpkox)_12_(H_2_O)_4_] that is present in the crystal of **4**·4EtOH·4­(*n*-hexane); Pr1 is the Pr^IV^ ion. O1X and O2X are the oxido and hydroxido oxygens, respectively. Diagnostic interatomic distances (Å): Pr1–O1X = 2.429(4), Pr2–O1X = 2.429(4), Pr3–O1X = 2.524(4), Pr1–O2X = 2.590(4), Pr2–O2X‴ = 2.455(4), Pr3–O2X = 2.478(4), Pr3–O41 = 2.475(6), Pr2–O41 = 2.495(6), Pr2″-O1 = 2.576(4), Pr3–O1 = 2.418(4), Pr3–N42 = 2.612(7), Pr­(2,3)-N_pyridyl_ = 2.733(5)–2.787(5), Pr2–N2 = 2.690(5), Pr3–N22″ = 2.627(5), Pr2″-O1 = 2.576(4), Pr3–O1 = 2.418(4), C6–N2 = 1.295(7), N2–O1 = 1.357(6), C26″-N22″ = 1.288(8), O21″-N22″ = 1.357(6), C46–N42 = 1.414(11), N42–O41 = 1.263(8), Pr···Pr = 3.963(4)–7.889(4). Selected bond angles (°): Pr–O1X-Pr = 110.7(2)–112.1(2), Pr–O2X-Pr = 104.7(1)–108.4(1), Pr2″–O1-Pr3 = 105.0(1), N42–Pr3–O41 = 28.6(2). Symmetry operations: ′2–*x*, 1/2–*y*, *z*; ″ 5/4-*y*, −3/4+*x*, 1/4–*z*; ‴ 3/4+*y*, 5/4–*x*, 1/4–*z*.

**Figure 5:**
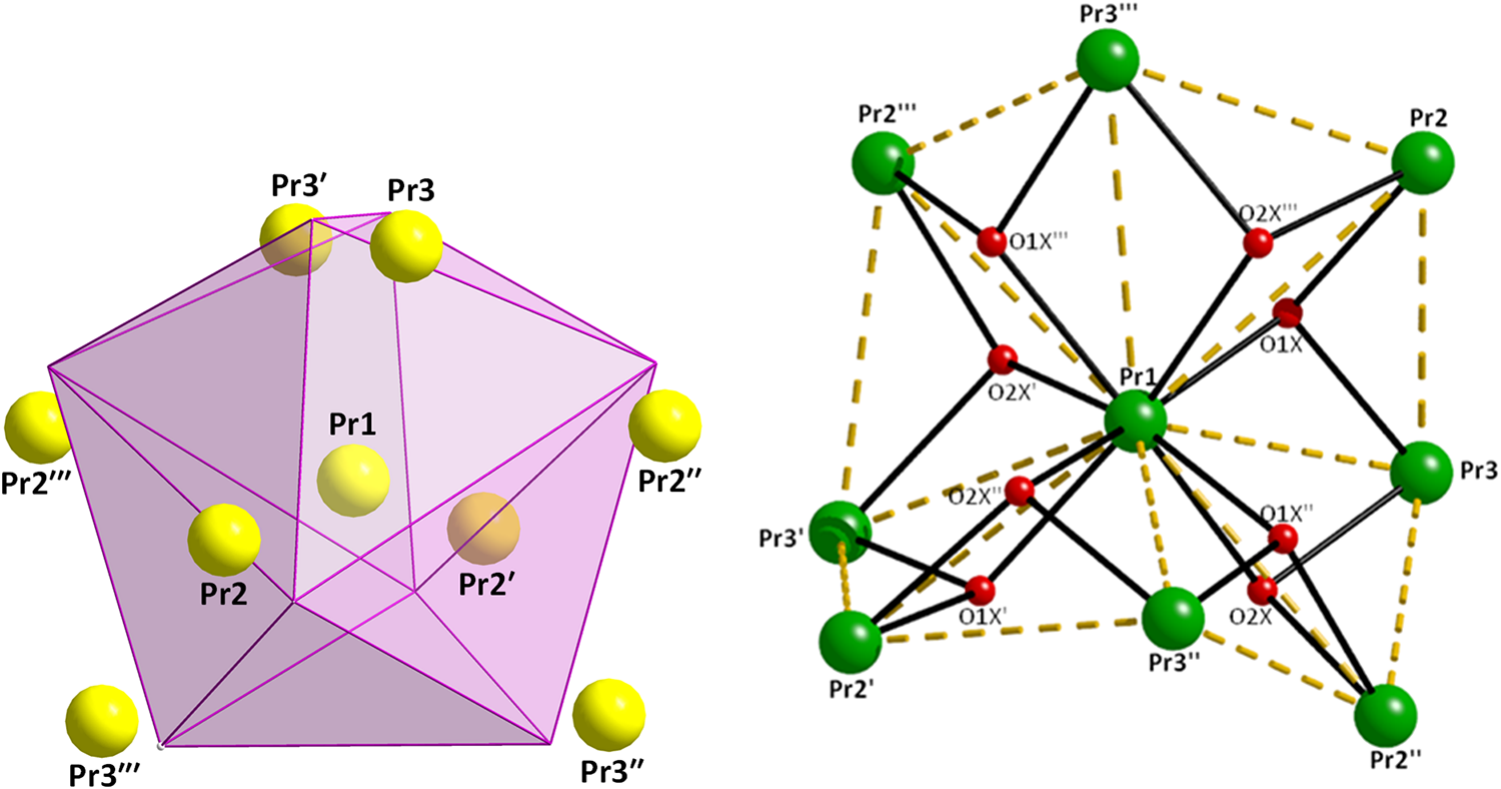
(Left) Representation of the triangular dodecahedral topology of the eight Pr^III^ ions (considering the tetravalent Pr1 as the central atom) in complex **4**·4EtOH·4­(*n*-hexane); points connected by the thin pink lines (which do not represent metal–metal bonds) define the vertices of the ideal polyhedron. (Right) The purely inorganic {Pr_8_^III^Pr^IV^(μ_3_-O)_4_(μ_3_–OH)_4_}^16+^ unit of the same complex. The symmetry operations are the same with those of Figure 4.

The central Pr^IV^ ion is connected to each peripheral Pr^III^ ion through one μ_3_–OH^–^ and one μ_3_-O^2–^ bridges; thus, its coordination sphere is {Pr1O_4_(OH)_4_}. The hard-base character (based on the HSAB model) of the oxido and hydroxido groups justifies the IV oxidation state of Pr1; Pr^IV^ is more oxophilic (i.e., harder acid according to the HSAB model) than Pr^III^ and the exclusive oxido-hydroxido environment favors the tetravalent state. The lower coordination number (i.e., 8) of Pr1 compared to the numbers of Pr2 and Pr3 (i.e., 9 and 10, respectively) is also an evidence for the description of Pr1 as tetravalent. However, in four of the all-Pr­(IV) structurally characterized complexes (*vide infra*), the metal ion adopts lower coordination numbers (4–6) with bulky ligands.^96–99^ Nevertheless, the coordination number 8 for Pr^IV^ has been crystallographically verified in complexes with an O_8_ environment.^100,101^ In the case of **4**, the coordination number 8 is probably due to the very small size of O^2–^ and OH^–^. The 2.1110 dpkox^–^ ligands link up Pr2 and Pr3 (and symmetry equivalents), the 3.2111 ligands connect two Pr2 and one Pr3 centers (and symmetry equivalents), and the 2.2110­(**B**) ones bridge Pr2 and Pr3 (and symmetry equivalents). Therefore, the coordination spheres of the trivalent metal ions are {Pr2O­(OH)­(O_oximato_)_3_(N_oximato_)­(N_pyridyl_)_3_} and {Pr3O­(OH)­(O_aquo_)­(O_nitrato_)_2_(O_oximato_)_2_(N_oximato_)_2_(N_pyridyl_)}. The coordination polyhedra (Figure S19) of Pr1, Pr2 and Pr3 are triangular dodecahedron (CShM = 1.541), spherical tricapped trigonal prism (CShM = 1.209) and sphenocorona (CShM = 4.658), respectively. The Pr1–O1X and Pr1–O2X bond lengths are 2.429(4) and 2.590(4) Å, respectively, typical for 8-coordinate Pr­(IV) complexes with praseodymium–oxygen bonds.^100,101^ Somewhat to our surprise, these bond lengths are comparable to those of the Pr2–O­(1X, 2X) and Pr3–O­(1X, 2X) ones that contain trivalent metal ions.

The oximato carbon–nitrogen and nitrogen–oxygen bond distances in the 2.1110 dpkox^–^ ligands are 1.288(8) and 1.357(6) Å, respectively; the corresponding lengths in the 3.2111 ligands are comparable, i.e., 1.295(7) and 1.357(6) Å. These values indicate a degree of delocalization for the oximato group, i.e., description as 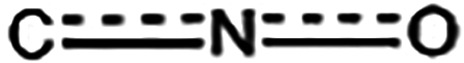, see structural study of **3**·2MeCN (*vide supra*). Of particular interest are the “oximato” carbon–nitrogen (C46–N42) and nitrogen–oxygen (N42–O41) bond distances in the 2.2110­(**B**) ligands, which are 1.414(11) and 1.263(8) Å, respectively. These ligands have the CNO fragment coordinated in the η^1^:η^2^:μ_2_ manner, with oxygen being the bridging atom. These carbon–nitrogen and nitrogen–oxygen bond distances are closer to those expected for single C–N and double N=O bonds, thus justifying the description of the CNO group as C-nitroso,^102,103^ and the correct representation of the coordination modes of these four, symmetry-equivalent dpkox^–^ ligands as 2.2110­(**B**), see Chart 4. Adopting this representation, complex **4**·4EtOH·4­(*n*-hexane) is the first lanthanoid complex with η^1^:η^2^:μ_2_-nitroso ligation. Generally, the anionic oxime (oximate) group is considered as a hybrid of the resonance forms **A** and **B** shown in Chart 6.^3^ Thus, it is evident that the CNO group in **3**, and in the 2.1110 and 3.2111 dpkox^–^ ligands of **4** has more **A** character, whereas this group in the 2.2110 ligands of the nonanuclear cluster has a more pronounced character of the resonance form **B**.

**Chart 6:**
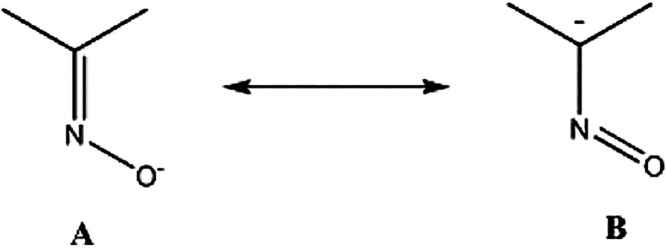
Two Resonance Forms of the 
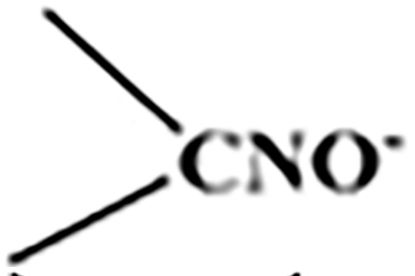
 Group

There are two, crystallographically independent weak intramolecular H bonds in **4**, with the hydroxido and aqua groups as donors and nitrato oxygens as acceptors. Their dimensions are: For O1W–H2­(O1W)···O63 (2–*x*, 1/2–*y*, *z*), O1W···O63 = 3.402(8) Å, H2­(O1W)···O63 = 2.52 Å and O1W–H2­(O1W)···O63 = 169.9°; and for O2X-H­(O2X)···O61 (2–*x*, 1/2–*y*, *z*), O2X···O61 = 2.833(5) Å, H­(O2X)···O61 = 1.91 Å and O2X-H­(O2X)···O61 = 148.1°, where O63 (not labeled in Figure 4) and O61 are free and coordinated nitrato oxygens, respectively. Neighboring {Pr_8_^III^Pr^IV^} molecules interact through C_aromatic_–H···O_nitrato_ H-bonding interactions (Figure S21a), creating a diamond-like lattice (Figure S21b).

Complex **4**·4EtOH·4­(*n*-hexane) is the third structurally characterized discrete Pr_9_ cluster. The previous ones are [Pr_9_^III^(OH)­(ClO_4_)­(Hpmp)_12_(H_2_O)_6_]­(ClO_4_)_13_,^104^ where Hpmp^–^ is the monoanion of *N*-piperidinomethane-1-phosphonic acid, and [Pr_9_^III^(OH)_10_(NO_3_)_8_(H_2_L)_8_]­(NO_3_),^105^ where H_2_L^–^ is the monoanion of 2-hydroxy-N-(2′-hydroxyethyl)-3-methoxybenzamide. The metal topology of the former was described as lotus-leaf-shaped and that of the latter as square antiprismatic with one metal ion occupying its center. Thus, **4** is the first mixed-valence Pr­(III)/Pr­(IV) cluster and has a new topology. Mixed III/IV valency has been reported once in the 3D compound {[Pr_2_^III^Pr_1.25_^IV^O­(OH)_3_(pydc)_3_]}*_n_*,^106^ where pydc^2–^ is the dianion of 2,5-pyridinedicarboxylic acid; this complex contains {Pr_6_(μ_3_-O)_2_(μ_3_–OH)_6_} building blocks of the bcu-x framework.

As mentioned in “Introduction section”, praseodymium can rarely be found in the oxidation states V^54,55,57^ and I.^58^ In addition to these extremely rare oxidation states, the IV level is also feasible for Pr. Its rareness has been explained in terms of the very high fourth ionization energy [the energy for the Pr^III^(g) → Pr^IV^(g) + e^–^(g) process] which is 3760 kJ mol^–1^, exceeding the sum of the first three ionization energies (∼3630 kJ mol^–1^). In a variety of extended binary and ternary oxides and fluorides (hard bases according to the HSAB model),^107–109^ e.g., PrO_2_, PrF_4_, APrF_5_, A_2_PrF_6_ and A_2_PrO_3_ (A = alkali metal ion), this barrier has been overcome. In molecular chemistry, Pr­(IV) complexes have long been deemed difficult to obtain. This situation has been changed dramatically since 2020, thanks to the pioneer work by Mazzanti,^96,97^ La Pierre^98^ and Zheng.^99^ Using the siloxide ^–^OSiPh_3_ ^96,97,99^ and the imidophosphorane {NP^*t*^Bu_3_}^−^ ^98^ ligands, these groups have achieved the synthesis and structural characterization of the solid complexes [Pr^IV^(OSiPh_3_)_4_(MeCN)_2_],^96^ [Pr^IV^(OSiPh_3_)_4_(OPPh_3_)­(MeCN)],^97^ [Pr^IV^(NP^*t*^Bu_3_)_4_][Bibr ref98] and [Pr^IV^(OSiPh_3_)_4_(L)],^99^ where L is the bidentate chelating ligand 4,4′-dimethoxy-2,2′-bipyridine, opening up new horizons in Pr­(IV) and generally in molecular Ln­(IV) chemistry. Additionally, the IV oxidation state for Pr has been observed and fully studied (CV, UV/vis/near-IR and X-band EPR techniques, and theoretical modeling) in solution,^110^ via oxidation of the anionic precursor K­[Pr^III^{NP­(1, 2-bis-^*t*^Bu-diamidoethane)­(NEt_2_)}_4_] with AgI at −35 °C. Two other homovalent Pr­(IV) complexes with different ligation are also known. These are the 1D coordination polymer {[Pr_2_^IV^(OH)_1.88_Cl_0.12_(L′)_6_]},^100^ where L is the monoanion of *N*-acetylanthranilic acid, and the cation in the polyoxometalate compound [Pr^IV^(NMP)_4_(H_2_O)_2_]^4+^[Ge^IV^Mo_12_^VI^O_40_]^4–^·2NMP·3H_2_O,^101^ where NMP is the neutral monodentate ligand *N*-methyl-2-pyrrolidone. Mixed-valence molecular Pr­(III/IV) clusters are unknown; the only molecular mixed-valence compound known is the 3D framework^106^ {[Pr_2_^III^Pr_1.25_^IV^O­(OH)_3_(pydc)_3_]}_n_ mentioned earlier.

### Mechanistic Insights

We briefly discuss here the proposed mechanism which is responsible for the formation of coordinated L^–^ in complexes **1**·3MeCN and **2**·3MeNO_2_. Other aspects of the mechanistic discussion are provided in the Electronic Supporting Information. Ketoximes have, in general, three nucleophilic centers, namely the N, O and C atoms of the oxime group (the third one is not applicable in the present study^3,111^ ). The O atom dominates the reactions under basic conditions, whereas the N atom is the strongest nucleophile at neutral media.^3^ The dpkoxH molecule bears two more nucleophilic sites, the N atoms of the 2-pyridyl rings, thus facilitating the formation of chelating rings with the participation of the oxime nitrogen. Taking into account the ability of dpkoxH to be hydrolyzed to dpk^26,74^ and the propensity of the latter to give its *gem*-diol form (dpk·H_2_O, Chart 3),^112–114^ both transformations in the presence of metal ions, Schemes S1 and S2 are proposed to account for the hydrolysis of dpkoxH to coordinated dpk under neutral (absence of Et_3_N) and basic (presence of Et_3_N) conditions, respectively. Both mechanistic schemes lead to the formation of the key-intermediate **VI**, shown in Scheme 3. The coordination mode of dpk in **VI** has been crystallographically confirmed in lanthanoid­(III) chemistry.^115^

**Scheme 3:**
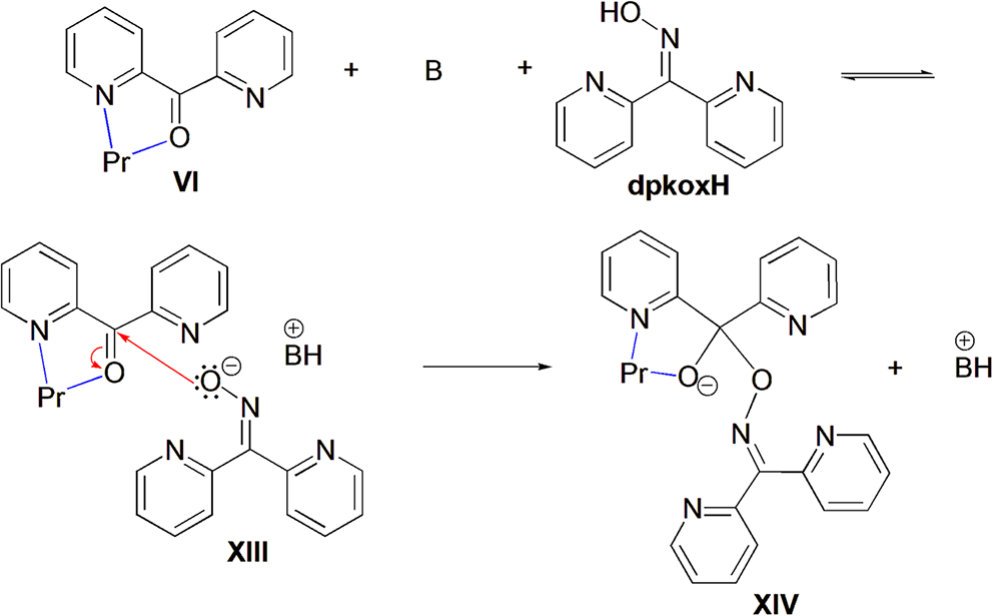
Proposed Mechanism for the Pr^3+^-Mediated Addition of dpkoxH on dpk to Produce the Species XIV Which is the “Precursor” That Leads to the Formation of the Complexes 1·3MeCN and 2·3MeNO_2_ ^a^,^b^

a The nitrato ligands have been omitted for clarity.
b For the identity of the chemical species that lead to **VI** and **XIII**, see Schemes S1, S2 and relevant discussion in the Electronic Supporting Information. B = base.

The final step of the reaction is the formation of the new ligand L^–^ (Scheme 3). The activated by the metal ion carbonyl function of the key-intermediate **VI** can be attacked by the nucleophiles present in the solution medium, namely H_2_O and the ketoxime. The former would lead to the corresponding ketone hydrate (the *gem*-diol form of dpk), whereas the latter would lead to the adduct LH. Due to the *a*-effect,^3^ the latter is much more nucleophilic than water, especially when deprotonated to the corresponding “oxide”. Accordingly, the mechanistic Scheme 3 can be envisaged for the last steps of the reaction leading to species **XIV**, which is the “precursor” for the two complexes **1**·3MeCN and **1**·3MeNO_2_. The proposed mechanistic scheme involves the following steps: (i) The first step involves the deprotonation of the ketoxime dpkoxH (p*K*_a_ = 7.79 ^116^) by either hydroxylamine (p*K*_a_ = 6.03) liberated during the hydrolysis of the ketoxime when the reaction is performed under neutral conditions, or the stronger base Et_3_N (p*K*_a_ = 10.78) when the reaction takes place under basic conditions. Thus, dpkoxH is in equilibrium with its corresponding “oxide” **XIII**, and (ii) the “oxide” **XIII** then attacks the carbonyl function of the species **VI** resulting in the “precursor” **XIV**.

### EPR Spectroscopy

The X-band EPR spectrum of a powdered sample of **4** at 4.2 K in the 0–3000 G range is shown in Figure 6. The spectrum is characterized by several resonances. The most prominent signals are a sharp peak at 630 G and a derivative feature at 1340 G. A closer examination reveals a multitude of weaker signals on the background. Such EPR signals are compatible with a half-integer spin system. The Pr^IV^ ion is a 4f^1^ ion with an effective *S*_eff_ = 1/2 Kramers’ doublet. The ^141^Pr nucleus (100% abundance) has a nuclear spin of *I* = 5/2. Mononuclear Pr­(IV) molecular coordination complexes^98,110^ and Pr^IV^-dopped oxide lattices^117,118^ exhibit EPR spectra characterized by a relatively strong hyperfine interaction with varying *g*- and *A*-anisotropy. From this point of view, the EPR behavior of an isolated Pr^IV^ ion deviates significantly from the behavior of an isolated 4f^1^ Ce^III^ ion for which the stable isotopes (^136/138/140/142^Ce > 99.8% abundance) have *I* = 0. In the present case, the Pr^IV^ center is part of a {Pr_9_} cluster. Although the exchange coupling between the ions is expected to be weak, the spectrum would be further affected by dipolar and hyperfine interactions from the neighboring paramagnetic Pr^III^ (4f^2^) ions as well, resulting in a complex EPR spectrum comprising a multitude of lines. Due to these complications, a quantitative analysis is premature at present. In any case, the observation of half-integer EPR signals is in agreement with a half-integer spin system and hence with a mixed-valence {Pr_8_^III^Pr^IV^} cluster.

**Figure 6:**
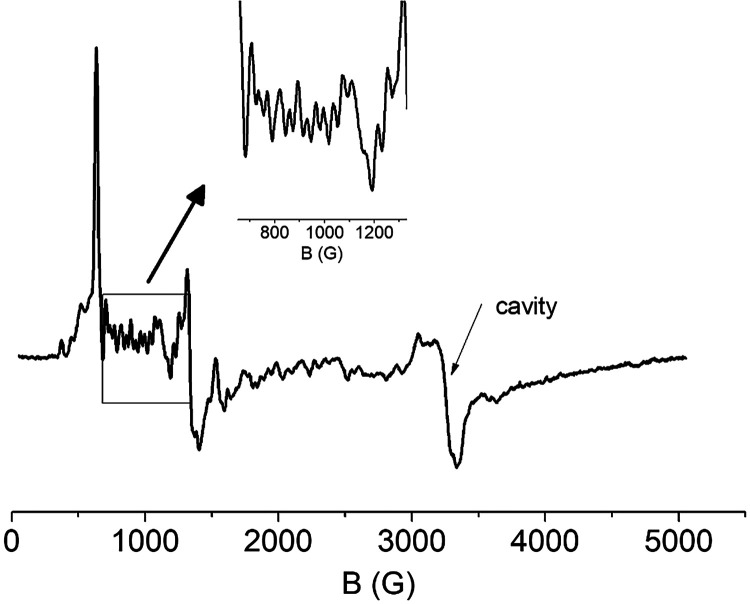
X-band EPR spectrum from a dried powdered sample of **4** at 4.2 K. EPR conditions: Modulation amplitude, 25 Gpp; microwave power, 30 mW; microwave frequency, 9.4 GHz. The arrow at ca. 3300 G indicates a derivative feature which is attributed to a cavity impurity. The inset shows a zoom of the spectrum in the region denoted by the rectangle.

## Concluding Comments

In conclusion, the detailed synthetic investigation of the Pr­(NO_3_)_3_·6H_2_O/dpkoxH reaction system has led to four interesting complexes (**1**–**4**), depending on the reaction conditions. Although mixed 3d or 4d/4f-metal complexes based on the dpkox^–^ ligand were reported ∼20 years ago^28–32^ (with emphasis on their magnetic behavior), the present compounds are the first *homometallic* complexes of dpkoxH with f-elements (4f, 5f), allowing us to complete the blank space of Pr in the “periodic table” of metals whose dpkoxH and/or dpkox^–^ complexes have been synthesized and characterized.^20^ Concerning the heterometallic *n*d/4f (*n* = 3, 4) dpkox^–^ - based complexes, the group of Ishida have prepared trinuclear {M^II^Ln_2_^III^} (M = Ni, Cu, Pd) clusters^28–32^ with the general formulas [NiLn_2_(hfac)_6_(dpkox)_2_(phen)], [NiLn_2_(hfac)_6_(py)_2_], [CuLn_2_(hfac)_6_(dpkox)_2_] and [PdLn_2_(hfac)_6_(dpkox)_2_], where phen is 1,10-phenanthroline, py is pyridine and hfac^–^ is the hexafluoroacetonato(−1) ligand. The molecules are almost linear (Ln···M···Ln = ∼ 175°). Each 2.1_2_1_1_1_1_1_2_ dpkox^–^ ligand bridges a Ln^III^M^II^ pair (the subscript 2 refers to Ln^III^ and the subscript 1 to M^II^). The central transition metal ion forms bonds to six N atoms (4 from the dpkox^–^ groups and two from the phen or py ligands) in the case of the {NiLn_2_} complexes (octahedral geometry), and to four N atoms (from the dpkox^–^ groups) in the case of the {M^II^Ln_2_^III^} (M = Cu, Pd) clusters (square planar geometry). Some Tb­(III)- and Dy­(III)-containing complexes are Single-Molecule Magnets (SMMs). According to our opinion, the most important chemical “messages” of this work are (a) A novel metal ion-assisted/promoted transformation of dpkoxH, either in the absence or presence of external base, has been discovered (compounds **1** and **2**) and the mechanism of formation of the unusual ligand L^–^ (which combines characteristics of di-2-pyridyl ketoxime and the *gem*-diol form of di-2-pyridyl ketone) has been explained. Thus, we believe that the results reported herein are a contribution to the reactivity of the coordinated oxime group, a currently “hot” topic in the frontier between inorganic and organic chemistry. (b) Compound **4**, which has a unique metal topology and core in 4f-metal chemistry, is the first mixed-valence Pr­(III/IV) cluster reported. From the synthetic viewpoint, the isolation of the {Pr^IV^Pr_8_^III^} complex shows that the combination of a very large concentration of OH^–^s and a polydentate N,O-based ligand in protic organic solvents, can create a “hard”-base environment around some Pr ions, favoring mixed-valency in 4f-metal, e.g., Ce, Pr and Tb, chemistry; and (c) The deprotonated oxime group is coordinatively flexible toward Ln ions; it is capable of forming a three-membered chelating ring, and can bridge either in a delocalized oximato or C-nitroso modes. A spectroscopic “message” of this work is that EPR spectroscopy is a useful tool for the study of Pr­(III/IV) mixed valency.

We believe that the described topic of research can give more results. We are currently aiming to expand the Pr­(III)/dpkoxH chemistry to heavier lanthanoids, e.g., Eu­(III), Tb­(III), Dy­(III) etc., as a means of isolating compounds with interesting optical and magnetic properties. Preliminary results have shown that the {Nd_4_^III^} analogue of **3** can be easily obtained and that the dpkox → L^–^ transformation in MeCN or MeNO_2_, in the absence or presence of a weak external base (e.g., Et_3_N), is a general trend across the lanthanoid series. Efforts are also in progress to isolate mixed-valence Ce­(III/IV) and Tb­(III/IV) complexes based on dpkox^–^, to discover new Ln­(III)-assisted/promoted reactivity of coordinated dpkoxH, and to extend the coordination chemistry of dpkoxH in uranyl and thorium­(IV) chemistry.

## Supplementary Material



## Notes

Crystallographic data, numerical data for H-bonding interactions, core of complex **3**, various structural and supramolecular figures, text with the description of the supramolecular structures of the complexes, mechanistic schemes and relevant discussion (PDF)

## ∥

Department of Materials Science & Technology, University of Crete, Voutes, 70013 Heraklion, Greece
