# Supplementary material for: Exploratory Synthetic Studies of the Praseodymium/Di-2-Pyridyl Ketoxime System Leads to Unusual Reactivity and Interesting New Molecules
Source: Inorg Chem. 2025 Dec 12;65(12):6385–401. doi: 10.1021/acs.inorgchem.5c04100 (PMC13040525; doi:10.1021/acs.inorgchem.5c04100)
Supplement: Supplementary file 1 [file ic5c04100_si_001.pdf]

ASSOCIATED CONTENT

Supporting Information

Exploratory Synthetic Studies of the Praseodymium/Di-2-Pyridyl Ketoxime System Leads to Unusual Reactivity and Interesting New Molecules

Christina Stamou,[a,$] Christina Polyzou,[a,$] Constantinos C. Stoumpos,[a,b] Catherine P. Raptopoulou,[c] Dionissios Papaioannou,[*a] Yiannis Sanakis,[*c] Vassilis Psycharis,[*c] and Spyros P. Perlepes[*a,d]

[a] Department of Chemistry, University of Patras, 26504 Patras, Greece

[b] Present address: Department of Materials Science & Technology, University of Crete, Voutes, 70013 Heraklion, Greece

[c] Institute of Nanoscience and Nanotechnology, NCSR "Demokritos", 15310 Aghia Paraskevi Attikis, Greece

[d] Institute of Chemical Engineering Sciences, Foundation for Research and Technology-Hellas (FORTH/ICE-HT), Platani, P.O. Box 1414, 26504 Patras, Greece

E-mail addresses for the corresponding authors
dapapaio@upatras.gr (D. P.)
i.sanakis@inn.demokritos.gr (Y. S.)
v.psycharis@inn.demokritos.gr (V. P.)
perlepes@upatras.gr (S. P. P.)
[$] C. S. and C. P. contributed equally to this paper.

Table S1. Crystallographic Data for Complexes 1·3MeCN, 2·3MeNO$_2$, 3·2MeCN and 4·4EtOH·4(n-hexane)

Parameter	1·3MeCN	2·3MeNO$_2$	3·2MeCN	4·4EtOH·4(n-hexane)
Formula	$C_{50}H_{41}Pr_2N_{17}O_{16}$	$C_{47}H_{41}Pr_2N_{17}O_{22}$	$C_{74}H_{68}Pr_4N_{24}O_{22}$	$C_{164}H_{188}Pr_9N_{40}O_{40}$
Fw	1417.82	1477.79	2209.15	4627.72
Crystal color	light green	light green	light green	light green
Crystal size (mm)	0.40x0.28x0.13	0.41x0.29x0.24	0.32x0.14x0.07	0.54x0.34x0.32
Crystal system	monoclinic	monoclinic	triclinic	tetragonal
Space group	$P2_1/n$	$P2_1/n$	*P-1*	$I4_1/a$
Temperature (°C)	-113	-113	-113	-83
Radiation (λ/Å)	Cu Kα (1.54178)	Cu Kα (1.54178)	Cu Kα (1.54178)	Cu Kα (1.54178)
a/Å	13.2674(2)	13.2338(2)	11.7520(2)	22.7784(4)
b/Å	16.0479(2)	16.0491(2)	13.2223(2)	22.7784(4)
c/Å	26.3924(5)	26.7752(5)	15.2775(2)	35.2950(6)
α/deg	90.00	90.00(1)	99.347(1)	90.00
β/deg	92.477(1)	94.625(1)	106.782(1)	90.00
γ/deg	90.00	90.00	109.922(1)	90.00
V/Å^3	5614.06(15)	5668.28(15)	2045.17(6)	18313.0(7)
Z	4	4	1	4
ρ_{calc}/g cm^{-3}	1.677	1.732	1.794	1.678
μ (mm^{-1})	13.874	13.851	18.695	18.670
F(000)	2824	2944	1092	9212
Reflections with $I>2\sigma(I)$	7947	8929	5953	6897
R_1 [a] [$I>2\sigma(I)$]	0.0665	0.0533	0.0520	0.0443
wR_2 [b] [$I>2\sigma(I)$]	0.1772 [c]	0.1482 [d]	0.1296 [e]	0.1226
Goodness of fit	1.07	1.05	1.06	1.04 [f]
CCDC number	2479197	2479198	2479199	2479200

[a] $R_1 = \Sigma(|F_0|-|F_c|)/\ \Sigma(|F_0|)$; [b] $wR_2 = \{\Sigma[w(F_0^2-F_c^2)^2]/\ \Sigma[w(F_0^2)^2]\}^{1/2}$, $w = 1/[\sigma^2(F_0^2)+(aP)^2+bP]$, where $P = [max(F_0^2)+2F_c^2]/3$; [c] a = 0.1167, b = 1.4563; [d] a = 0.1061, b = 6.5312; [e] a = 0.0765, b = 4.7344; [f] a = 0.0700, b = 60.9033.

Table S2. H-bonding Geometry (Å, °) for Complex 2·3MeNO$_2$

D-H···A	D-H	H···A	D···A	D-H···A
		Intramolecular H-bonding interactions		
C8-H8···N5	0.99(6)	2.63(6)	3.599(8)	166(4)
C38-H38···N35	0.96(8)	2.74(7)	3.589(7)	147(6)
		Intralayer H-bonding interactions		
C43-H43···O70^{i}	0.95	2.45	3.382(7)	166
C15-H15···O63^{ii}	0.95(7)	2.59(7)	3.457(7)	152(5)
		Interlayer H-bonding interactions		
C2-H2···O69^{iii}	0.95	2.58	3.343(7)	137
		H-bonding interactions with the lattice MeNO$_2$ molecules		
C19-H19···O81^{ii}	0.95	2.43	3.074(11)	125
C10-H10···O85^{iii}	1.02(8)	2.54(8)	3.556(9)	173(6)
C13-H13···O83^{iv}	1.07(7)	2.51(7)	3.361(12)	136(5)

Symmetry codes: ^{i}5/2-*x*, ½+*y*, ½-*z*; ^{ii}1+*x*, *y*, *z*; ^{iii}*x*, -1+*y*, *z*; ^{iv}3/2-*x*, -1/2+*y*, ½-*z*. D = donor, A = acceptor.

Table S3. H-bonding Geometry (Å, °) for Complex 1·3MeCN

D-H···A	D-H	H···A	D···A	D-H···A
		Intramolecular H-bonding interactions		
C9-H9···N3	0.95	2.77	3.685(10)	162
C36-H36···O64	0.95	2.55	3.179(9)	124
		Interdimer H-bonding interactions		
C4-H4···O66^{i}	0.95	2.69	3.469(11)	140
C2-H2···O75^{i}	0.95	2.57	3.402(9)	147
		Intralayer H-bonding interactions		
C37-H37···O73^{ii}	0.95	2.40	3.281(10)	155
		Interlayer H-bonding interactions		
C49-H49···O63^{iii}	0.81(9)	2.69(9)	3.381(10)	145(8)
		H-bonding interactions with the lattice MeCN molecules		
C7-H7···N82^{iv}	0.95	2.55	3.494(13)	176
C82-H82B···O61^{ii}	0.98	2.52	3.468(3)	164
C84-H84B···O66^{iii}	0.98	2.39	3.102(4)	129

Symmetry codes: ^{i}1-*x*, -*y*, 2-*z*; ^{ii}1-*x*, 1-*y*, 2-*z*; ^{iii}3/2-*x*, -1/2+*y*, 3/2-*z*; ^{iv}3/2-*x*, 1/2+*y*, 3/2-*z*. C82 and C84 belong to MeCN molecules. D = donor, A = acceptor.

Table S4. H-bonding Geometry (Å, °) for Complex 3·2MeCN

D-H···A	D-H	H···A	D···A	D-H···A
		Intracluster H-bonding interactions		
O51-H(O51)···N3^{i}	0.95(12)	1.80(12)	2.728(7)	164(11)
C21-H21···O21^{i}	0.95	2.46	3.093(9)	124
		Intralayer H-bonding interactions		
C10-H10···O33^{ii}	0.95	2.53	3.363(10)	146
		Interlayer H-bonding interactions		
C31-H31···N13^{iii}	0.95	2.62	3.438(9)	144
C50-H50···O43^{iv}	0.95	2.66	3.435(13)	139
		H-bonding interactions with the lattice MeCN molecules		
C2S-H(C2S)···O41^{v}	0.98	2.51	3.215(11)	129

Symmetry codes: ^{i}2-*x*, 1-*y*, 1-*z*; ^{ii}1+*x*, *y*, 1+*z*; ^{iii}1-*x*, -*y*, -*z*; ^{iv}3-*x*, 2-*y*, 1-*z*. C2S belongs to the MeCN molecule. D = donor, A = acceptor.

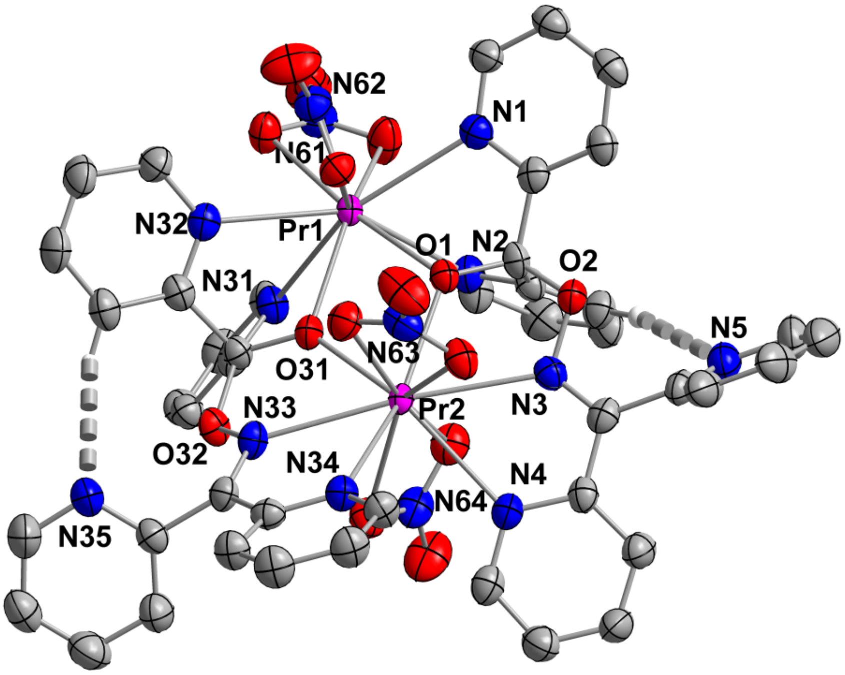

Figure S1. An ORTEP-type presentation of the structure of molecule **2**. Dashed light gray lines indicate intramolecular H-bonding interactions. Only the H atoms involved in the interactions are shown for clarity. Numerical data are given in Table S2.

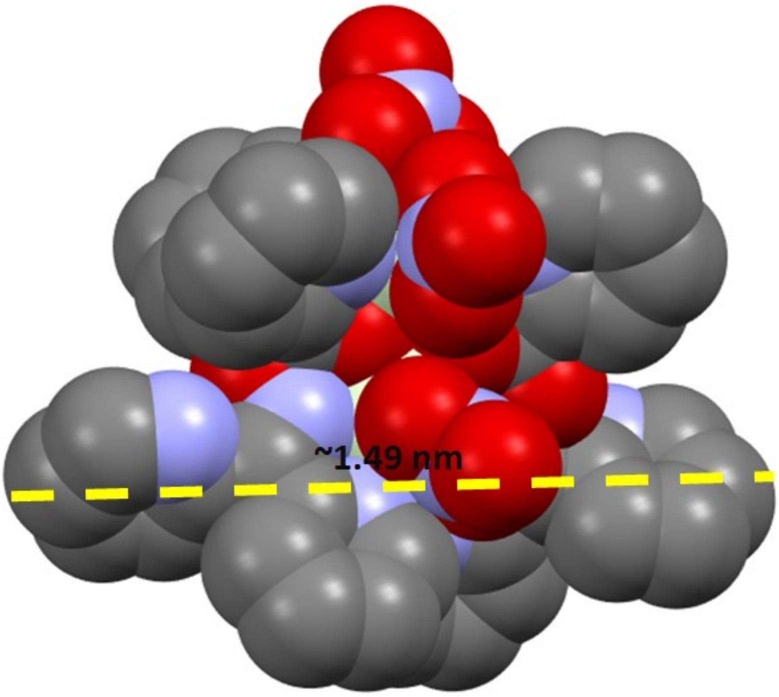

Figure S2. Space-filling diagram of **2**.

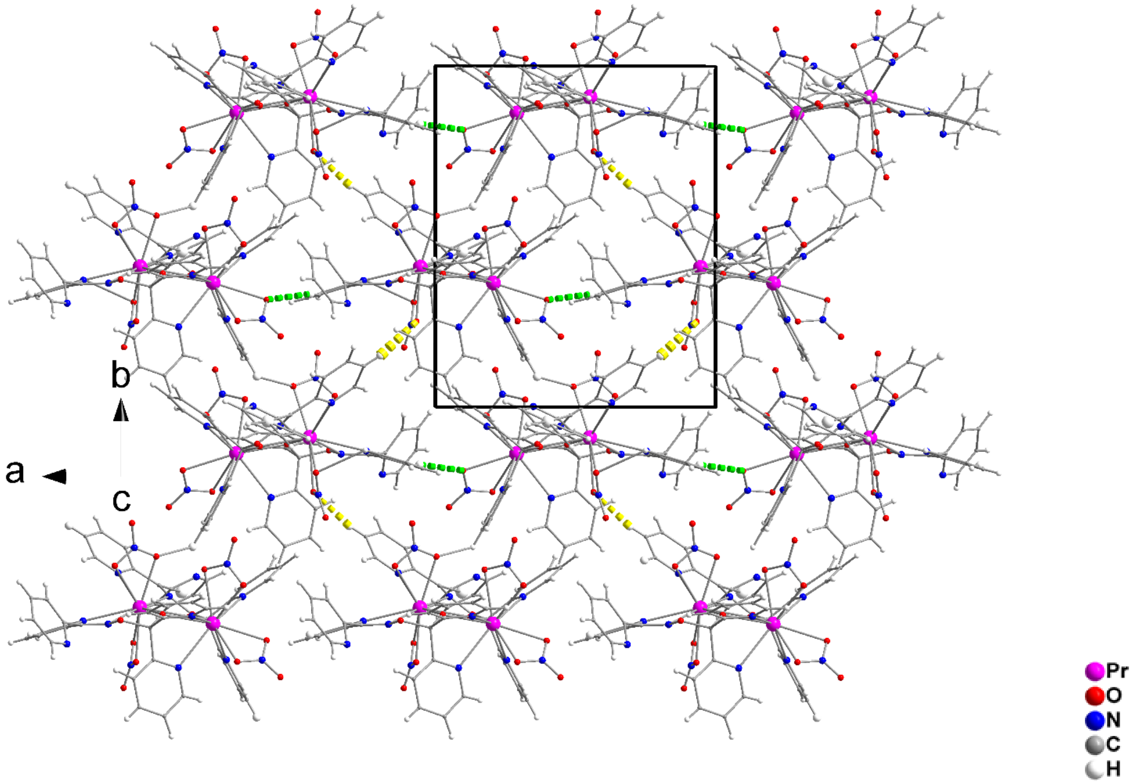

Figure S3. A layer of molecules **2** parallel to the (001) planes. Dashed yellow and light green lines indicate the C43-H43···O70 and C15-H15···O63 H-bonding interactions, respectively. Only the H atoms involved in these interactions are shown for clarity. Numerical data are given in Table S2.

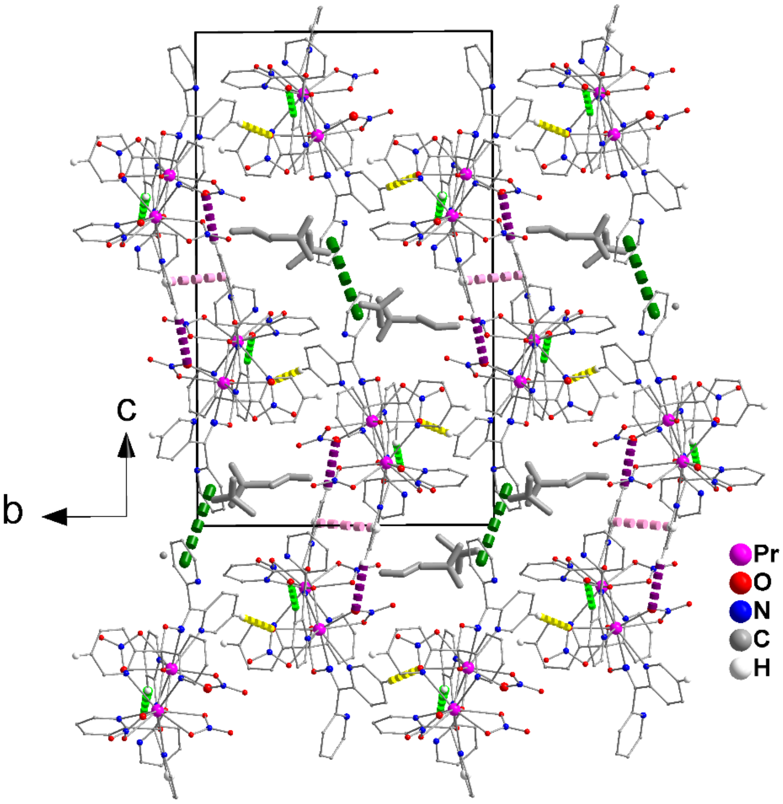

Figure S4. The 3D arrangement of molecules **2** and $MeNO_2$ molecules through C2-H2···O69 H bonds (indicated with dashed violet lines) and π-π interactions. The lattice $MeNO_2$ molecules are shown in gray color and capped pick style. Only the H atoms involved in the interactions are shown. Numerical data are given in Table S2.

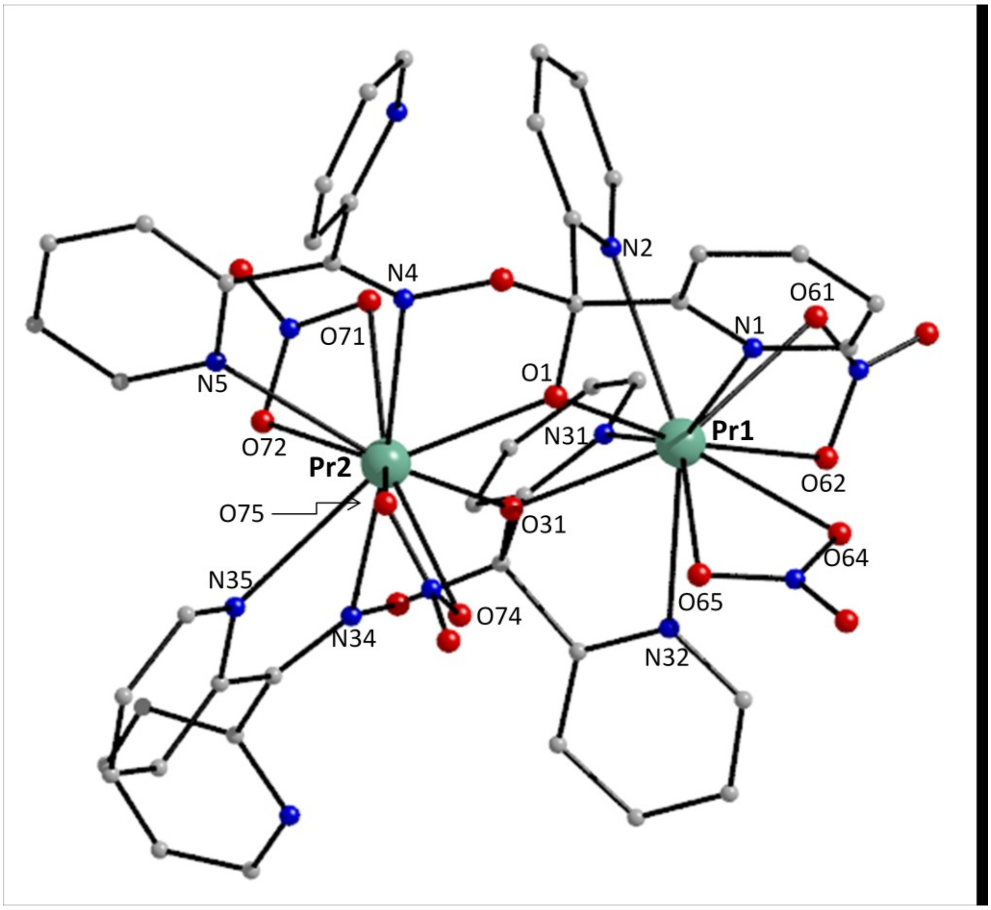

Figure S5. The structure of the molecule $[Pr_2(NO_3)_4(L)_2]$ that is present in the crystal of **1**·3MeCN. Pr-$O_{alkoxido}$ = 2.386(4)-2.430(4) Å, Pr-$O_{nitrato}$ = 2.544(5)-2.591(5) Å, Pr-$N_{pyridyl}$ = 2.650(6)-2.739(5) Å, Pr2-N4 = 2.671(6) Å, Pr2-N34 = 2.685(6) Å, Pr1···Pr2 4.046(1) Å, O61-Pr1-O62 = 48.5(2)°, O71-Pr2-O72 = 50.5(2)°, O1-Pr1-O62 = 169.6(2)°, N4-Pr2-N34 = 173.2(2)°, Pr1-O1-Pr2 = 112.9(2)°, Pr1-O31-Pr2 = 115.4(2)°.

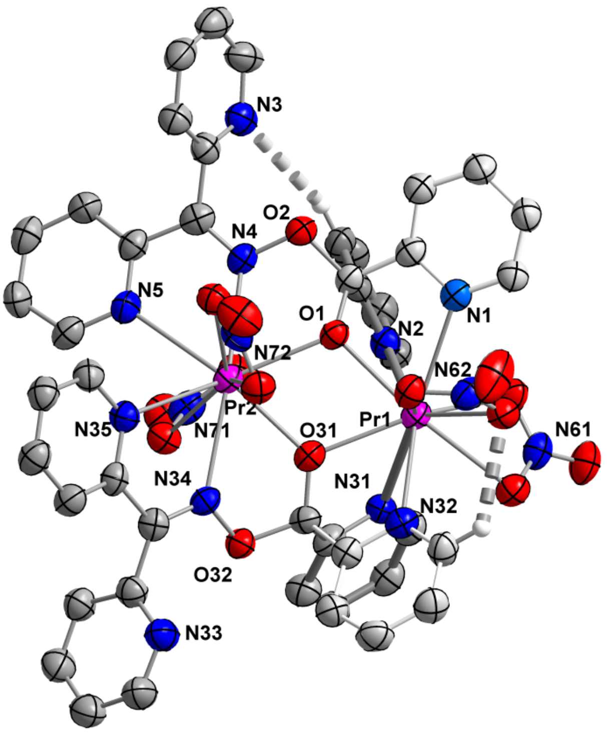

Figure S6. An ORTEP-type presentation of the structure of the molecule **1**. Dashed light gray lines indicate intramolecular H-bonding interactions. Only the H atoms involved in the interactions are shown for clarity. Numerical data are given in Table S3.

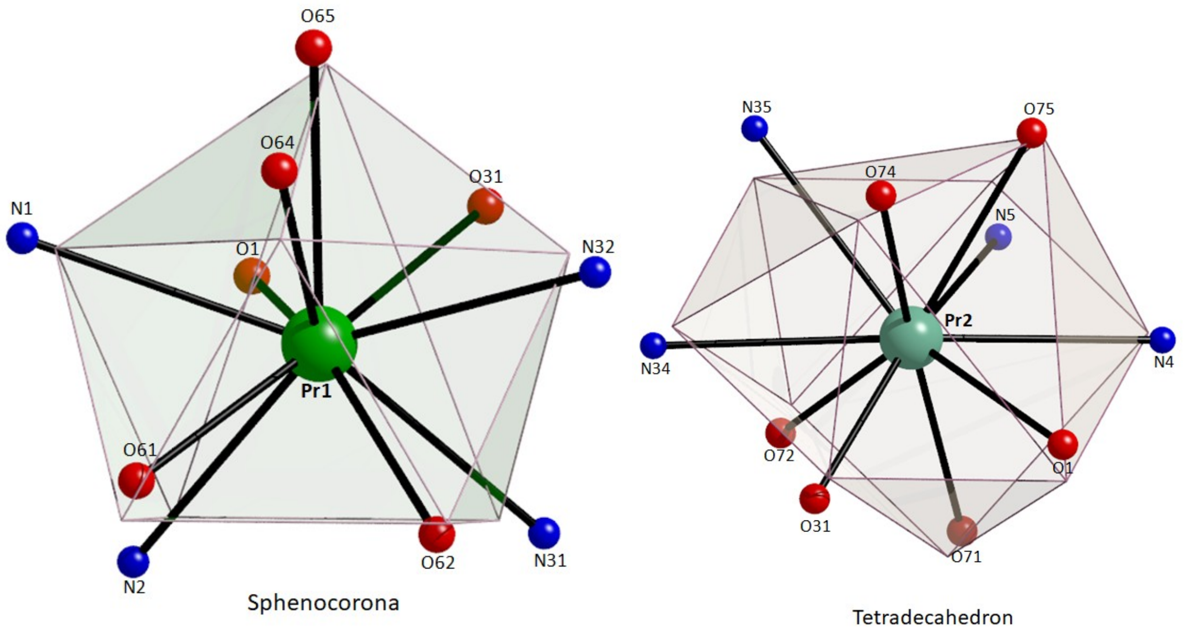

Figure S7. The coordination polyhedra of Pr1 and Pr2 in the structure of **1**·3MeCN. The CShM values are 3.562 and 3.839 for Pr1 and Pr2, respectively. The very small spheres represent the vertices of the ideal polyhedra.

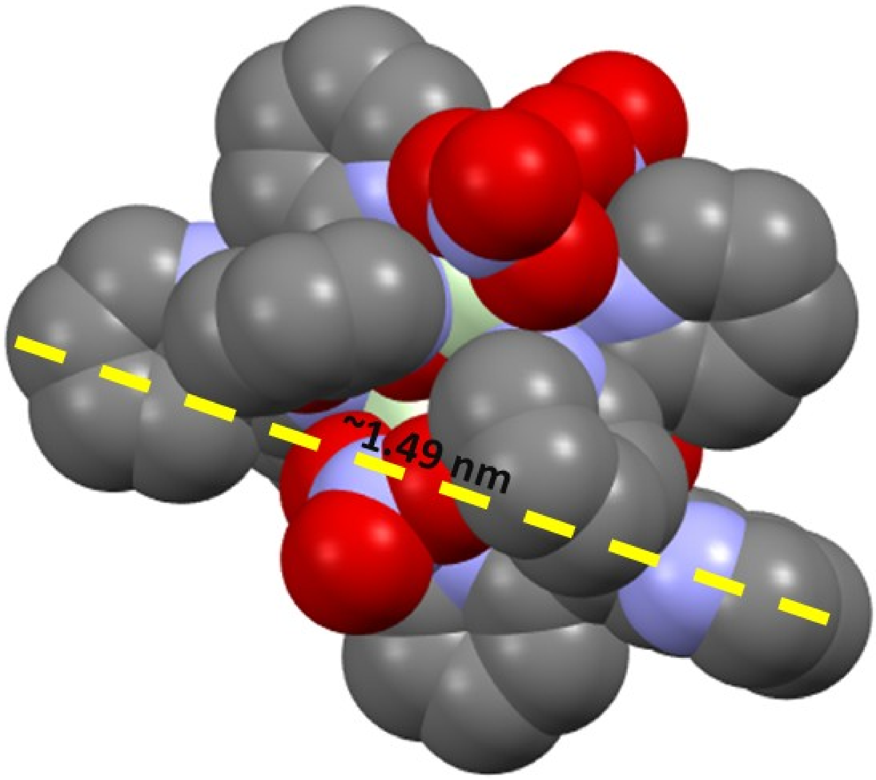

Figure S8. Space-filling diagram of **1**.

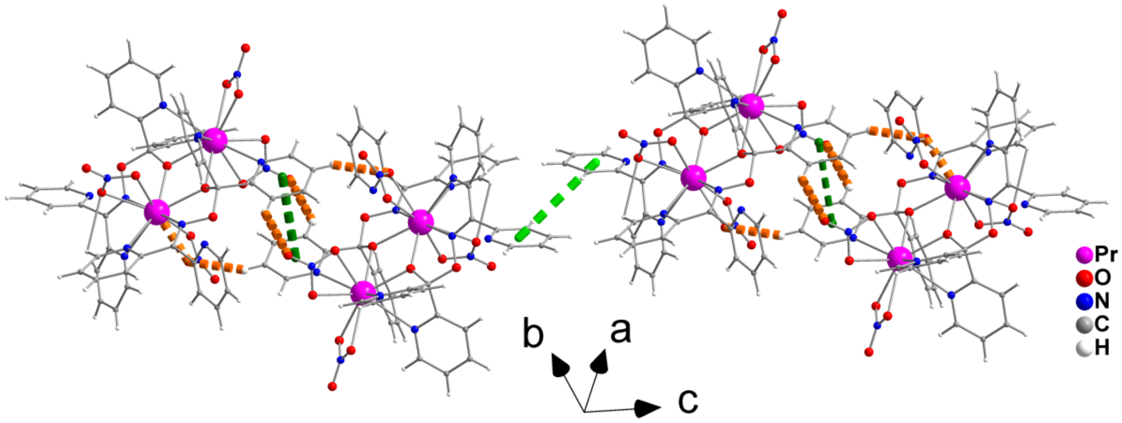

Figure S9. A chain of molecules **1** parallel to the *c* axis. Dashed orange lines indicate the C4-H4···O66 and C2-H2···O75 H-bonding interactions. Dark green and light green lines indicate π-π interactions between pyridyl rings. Numerical data are given in Table S3.

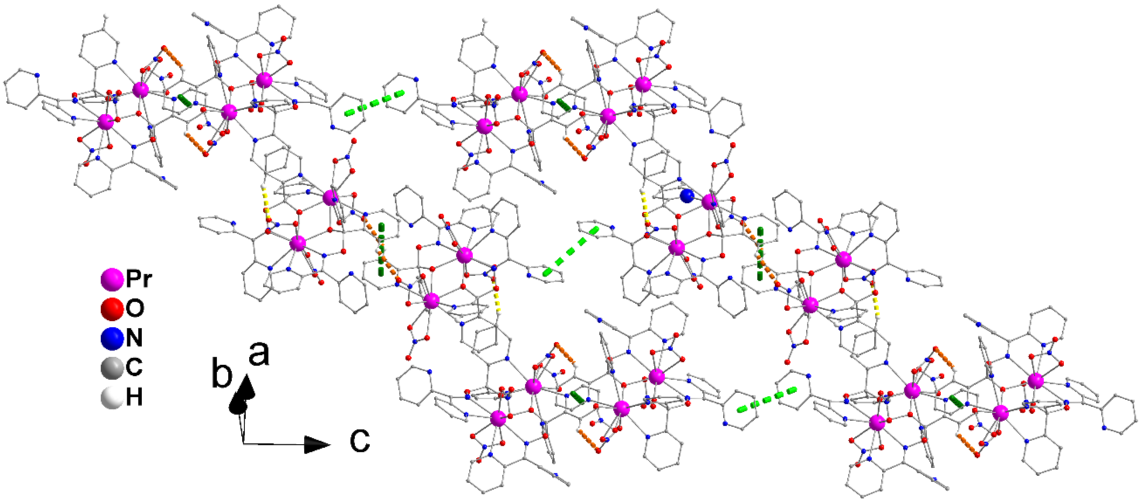

Figure S10. A layer of molecules **1** parallel to the (1-10) planes. The color coding of the interactions is as in Figure S9. The additional C37-H37···O73 H-bonding interactions are represented with dashed yellow lines. Only the H atoms involved in these interactions are shown for clarity. Numerical data are given in Table S3.

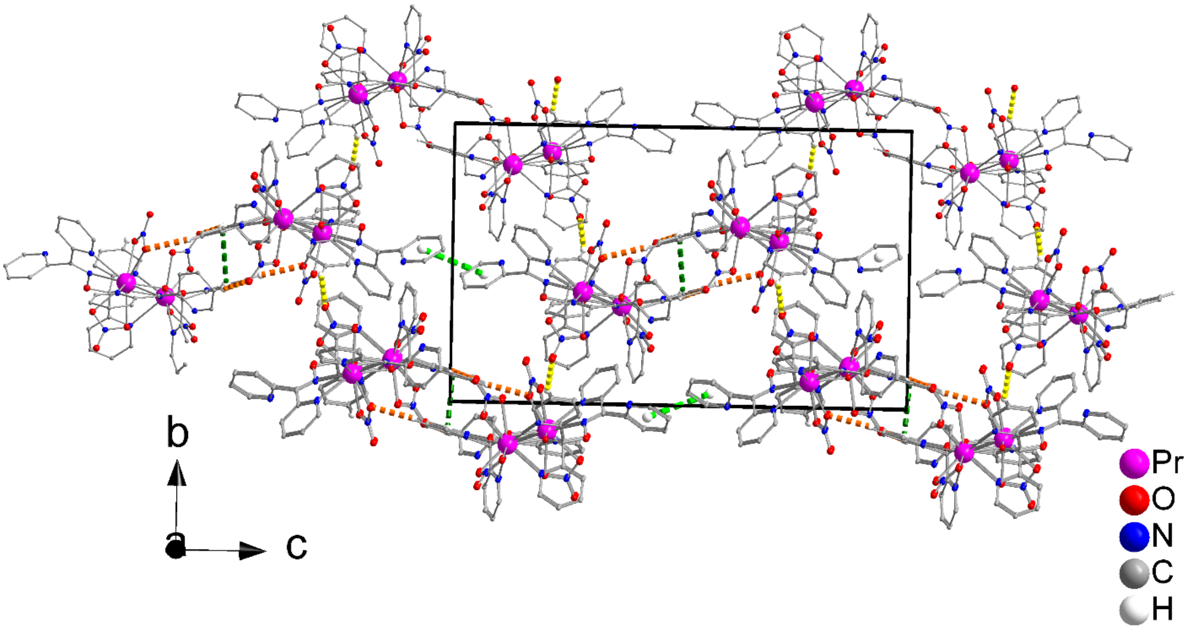

Figure S11. The 3D arrangement of molecules **1** built through the additional C49-H49···O63 H bonding interactions (indicated with dashed violet lines). The color coding of the other interactions is as in Figures S9 and S10. The light gray rods shown in the voids of the structure represent the lattice MeCN molecules. Only the H atoms involved in the interactions are shown. Numerical data are given in Table S3.

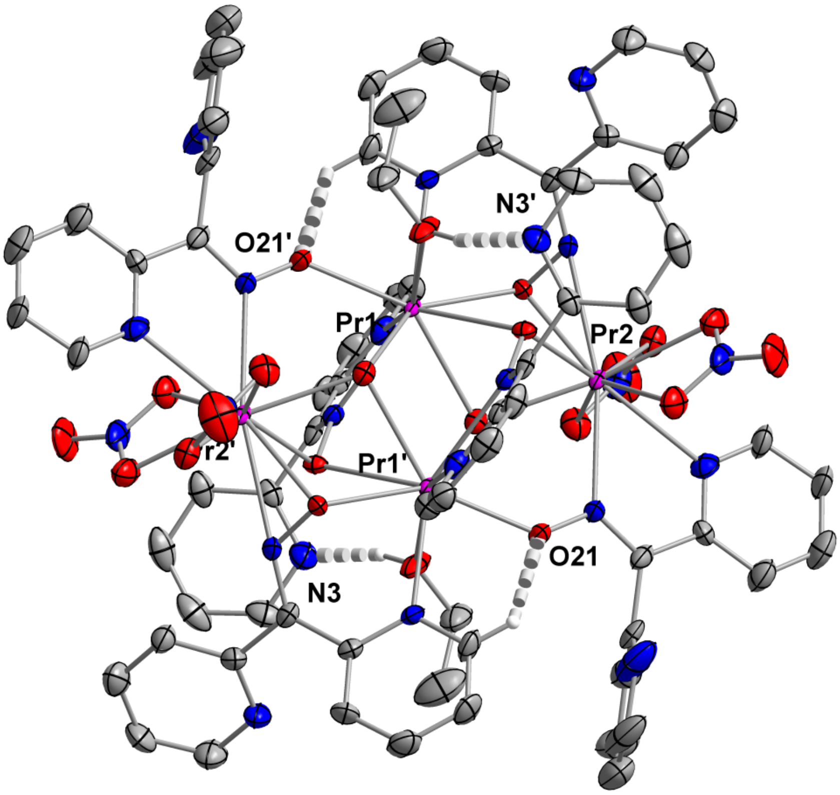

Figure S12. An ORTEP-type presentation of the structure of the molecule **3**. The symmetry operation is as in Figure 3. Dashed light gray lines indicate intramolecular H-bonding interactions. Only the H atoms involved in the interactions are shown for clarity. Numerical data are given in Table S4.

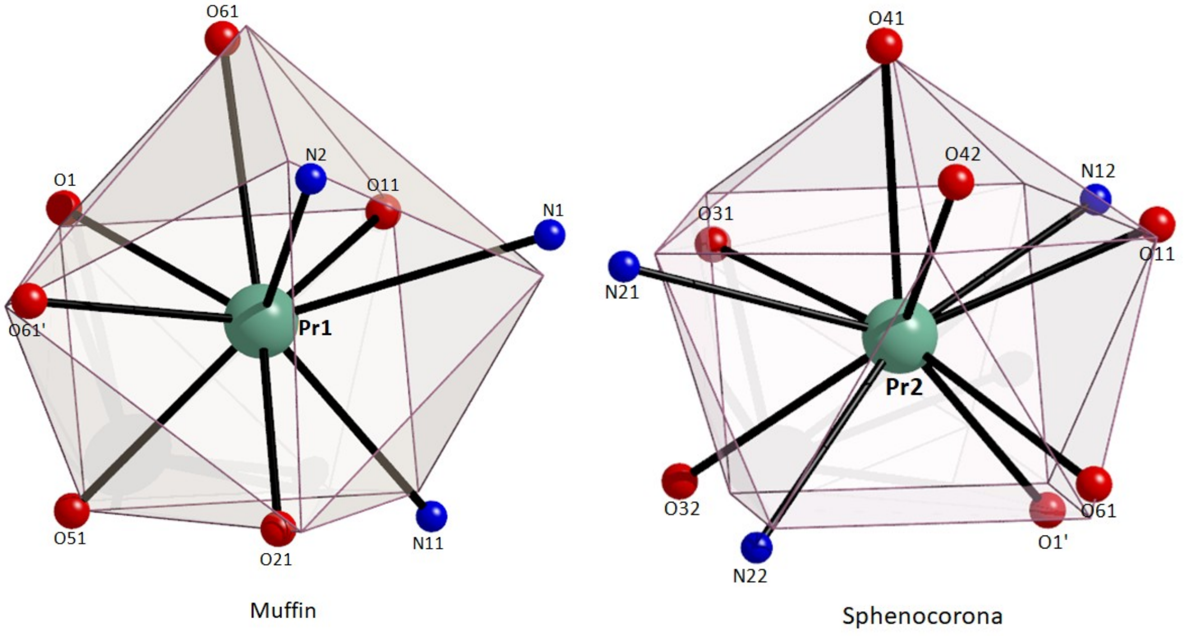

Figure S13. The coordination polyhedra of crystallographically independent ions Pr1 and Pr2 in the structure of **3**·2MeCN. The CShM values are mentioned in the text. The very small spheres represent the vertices of the ideal polyhedra. Symmetry operation: [i]2-*x*, 1-*y*, 1-*z*.

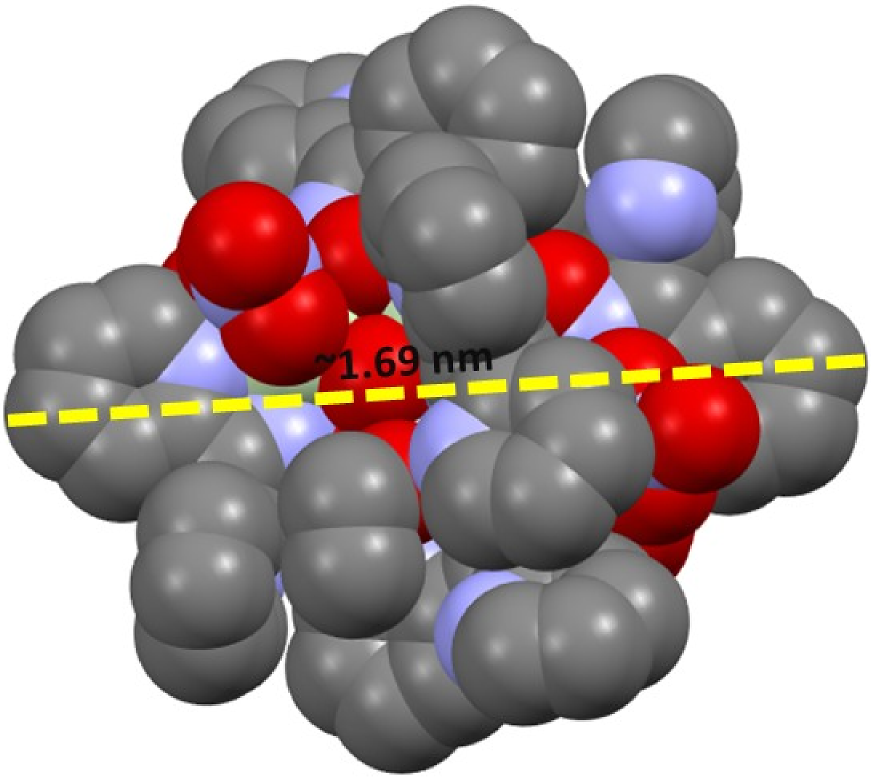

Figure S14. Space-filling diagram of **3**.

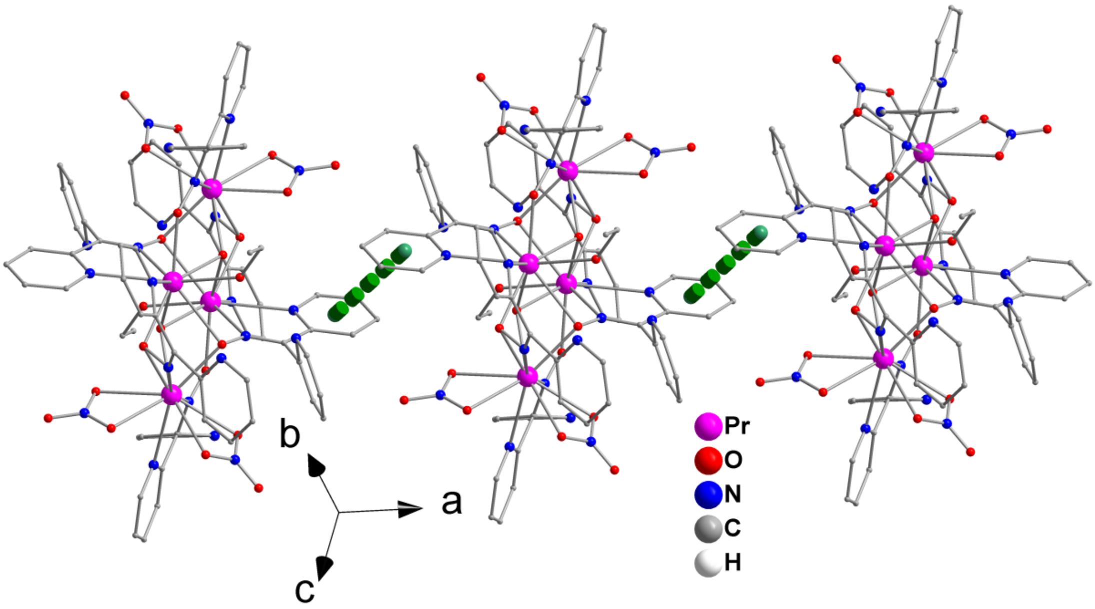

Figure S15. A chain of molecules **3** parallel to the *a* axis. Dashed dark green lines indicate π-π interactions between pyridyl rings. Numerical data are given in Table S4.

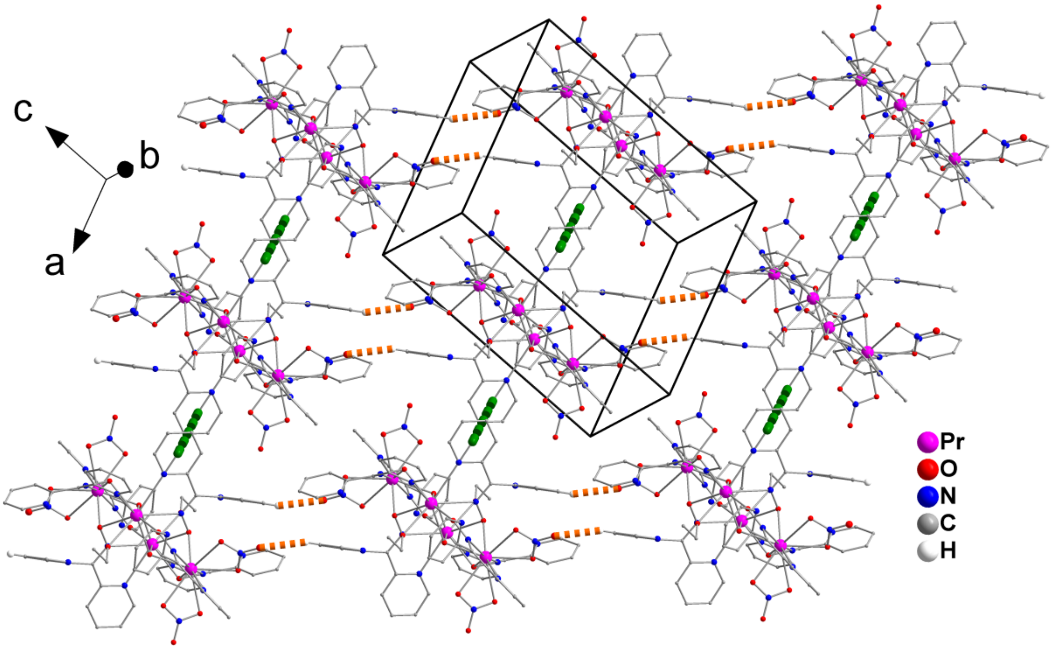

Figure S16. A layer of molecules **3** parallel to the (010) planes. The color coding of bonds is as in Figure S9. The additional C10-H10···O33 H-bonding interactions are represented with dashed orange lines. Only the H atoms involved in these interactions are shown for clarity. Numerical data are given in Table S4.

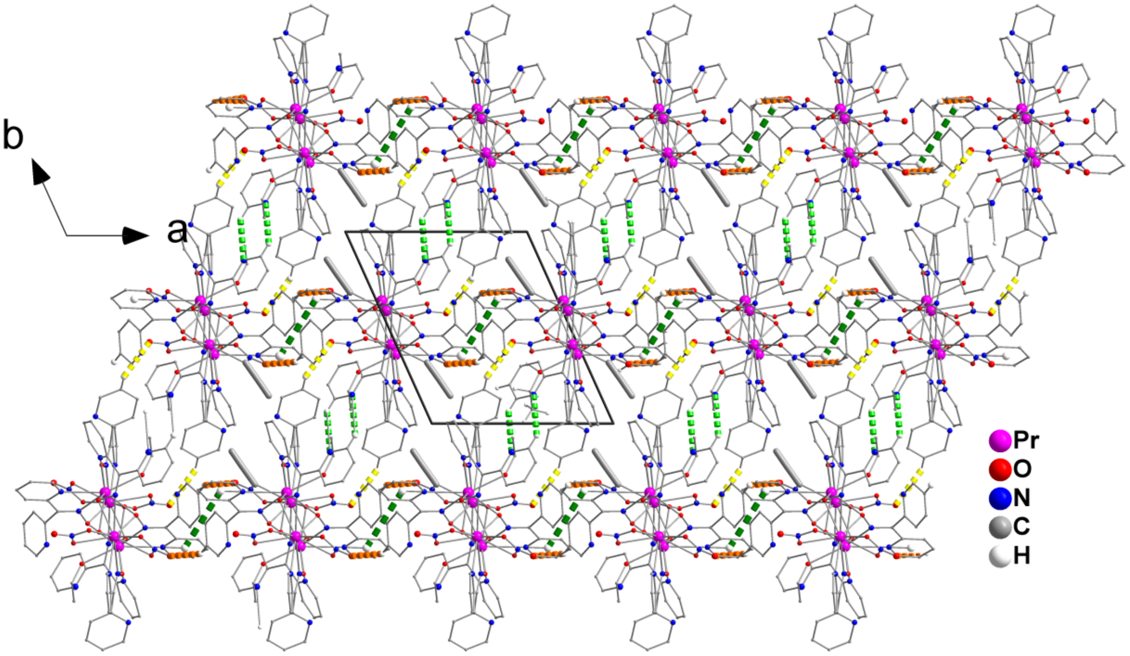

Figure S17. The 3D arrangement of molecules **3** built through the additional C31-H31···N13 and C50-H50···O43 H-bonding interactions, indicated with dashed light green and yellow lines, respectively. The coloring code of the other interactions is as in Figures S15 and S16. The lattice MeCN molecules are presented as gray rods. Only the H atoms involved in the interactions are shown. Numerical data are given in Table S4.

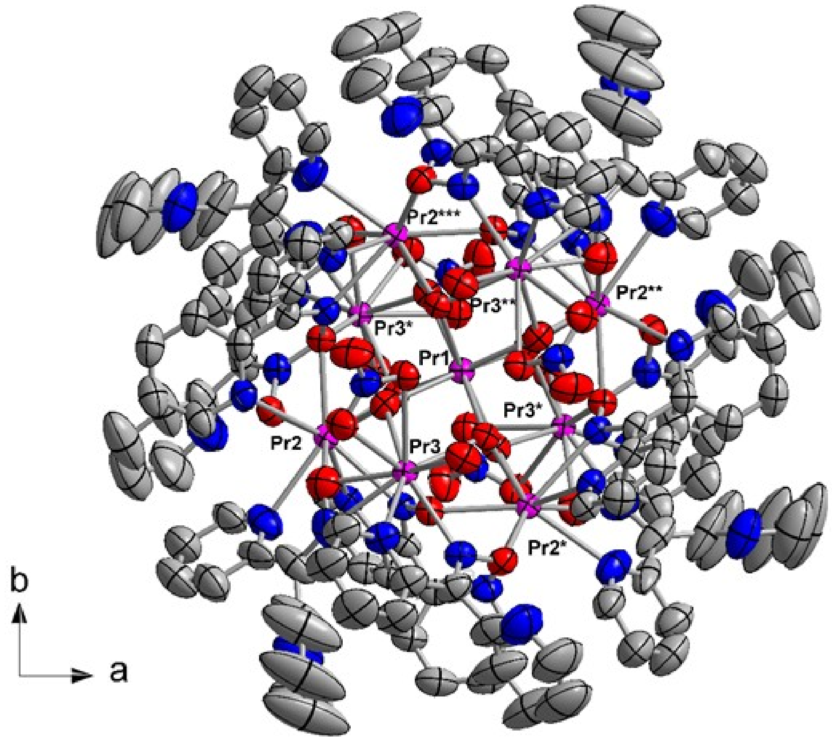
Pr2***
Pr3**
Pr2**
Pr3*
Pr1
Pr3*
Pr2
Pr3
Pr2*
b
a

a)

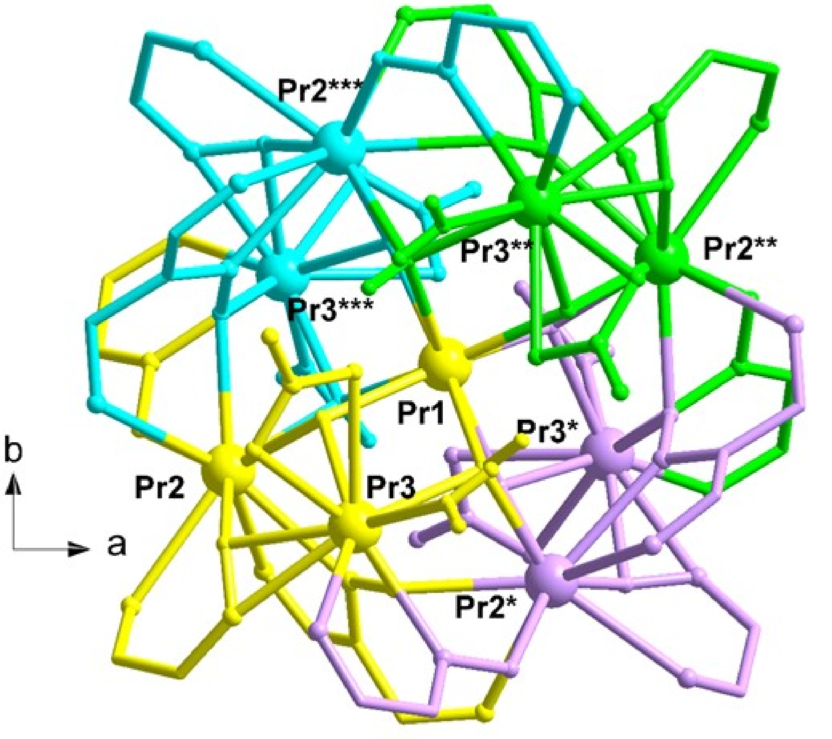
Pr2***
Pr3**
Pr2**
Pr3***
Pr1
Pr3*
Pr2
Pr3
Pr2*
b
a

b)

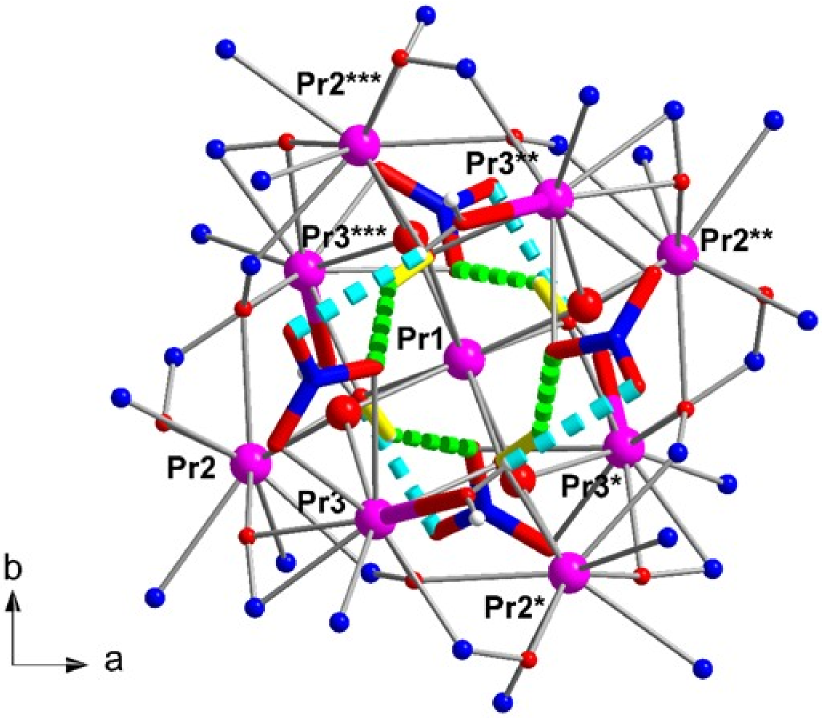
Pr2***
Pr3**
Pr3***
Pr2**
Pr1
Pr2
Pr3
Pr3*
Pr2*
b
a

c)

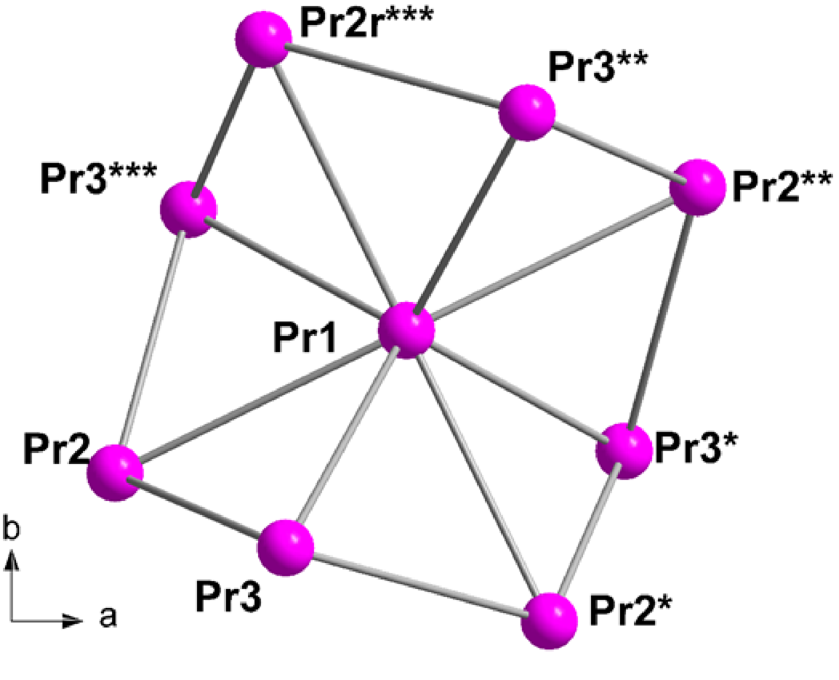
Pr2r***
Pr3**
Pr3***
Pr2**
Pr1
Pr2
Pr3*
b
a
Pr3
Pr2*

d)

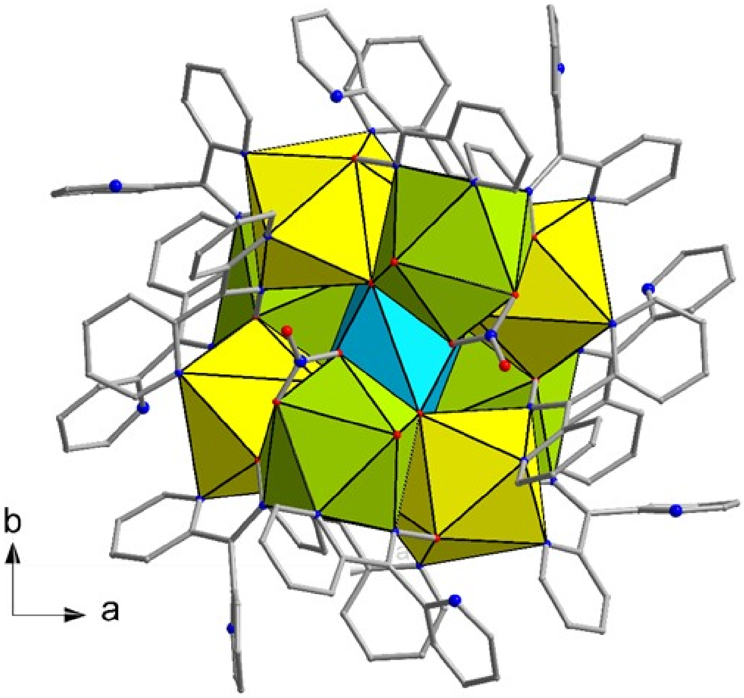

e)

Figure S18. a) A partially-labelled ORTEP-type presentation of the structure of molecule **4** shown along the –*c* axis. Symmetry codes: Pr1, Pr2, Pr3 *x*, *y*, *z*;* 5/4-*y*, -3/4+*x*, ¼-*z*;** 2-*x*, ½-*y*, *z*; ***3/4+*y*, 5/4-*x*, ¼-*z*. H atoms are not shown for clarity. b) A same view (as in a above) of **4**, without the pyridyl rings, in capped rod-type presentation with color differentiation of the different symmetric parts of the molecule. Color codes: 0*x*, *y*, *z* (yellow); *5/4-*y*, -3/4+*x*, ¼-*z* (violet); **2-*x*, ½-*y*, *z* (light green); ***3/4+*y*, 5/4-*x*, ¼-*z* (cyan). c) A same view (as in a and b above) showing only the donor atoms of the metal cations. Thick dashed light green and cyan lines indicate the O1W-H2(O1W)···O63 and O2X-H(O2X)···O61 H bonds, respectively; for their symmetry codes and numerical data, see text. Thick yellow and red-pink lines indicate the O2X-H(O2X) and Pr1-O1W bonds. Large red spheres represent the $O1X^{2-}$ ions. d) A view of the molecule showing only the metal ions which form eight triangles with the Pr^{IV} ion (Pr1) as common vertex; each triangle has two common sides with the neighboring triangles. The Pr^{IV}···Pr^{III} distances are in the range 4.963(4)-4.110(1) Å; and e) The molecule in polyhedral presentation; the Pr1, Pr2 and Pr3 polyhedra are presented with cyan, yellow and lime colors, respectively.

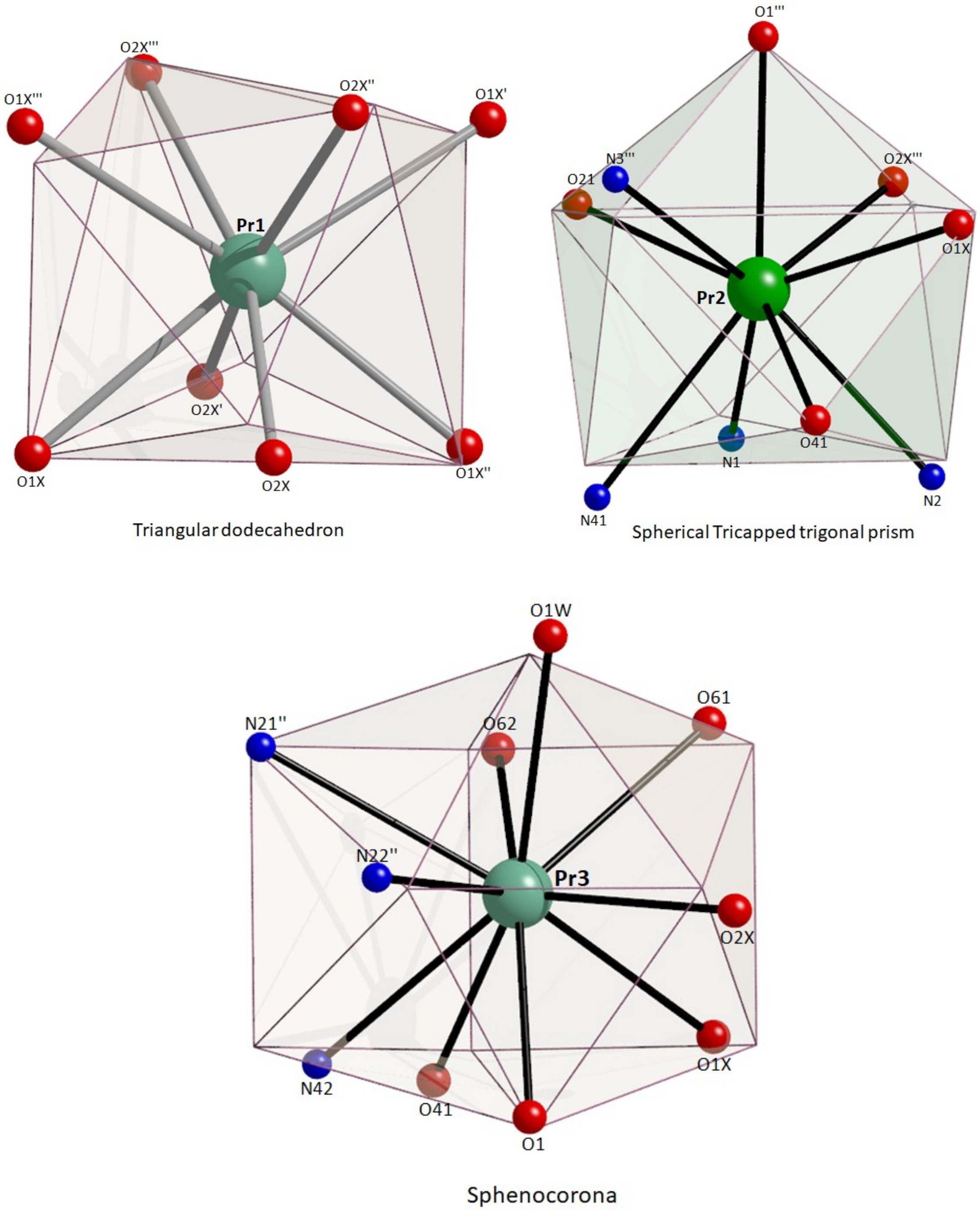

Figure S19. The coordination polyhedra of Pr1, Pr2 and Pr3 in the structure of **4**·4EtOH·4(n-hexane). The CShM values are 1.541, 1.209 and 4.658, respectively. The very small spheres represent the vertices of the ideal polyhedra.

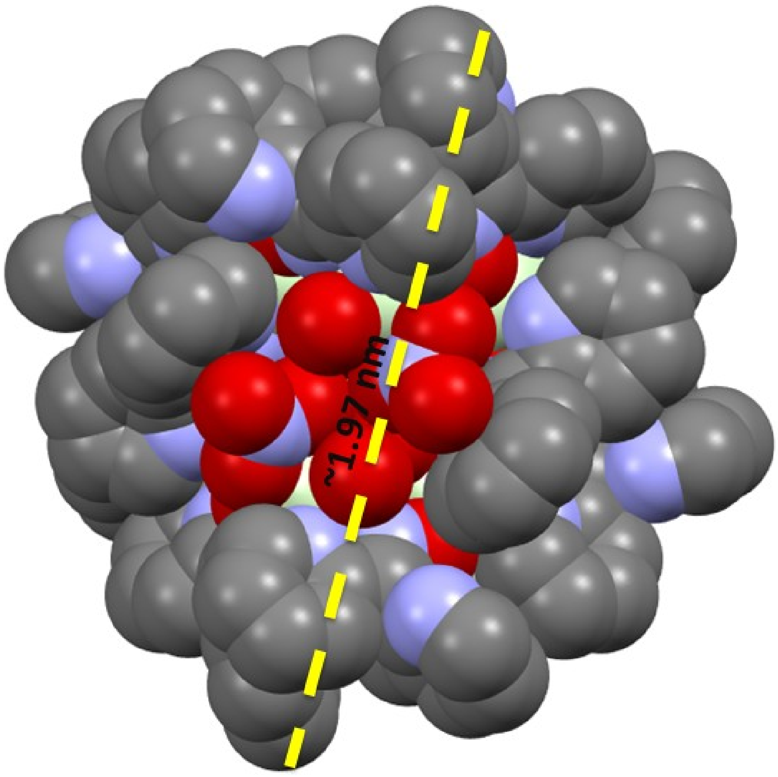

Figure S20. Space-filling diagram of **4**.

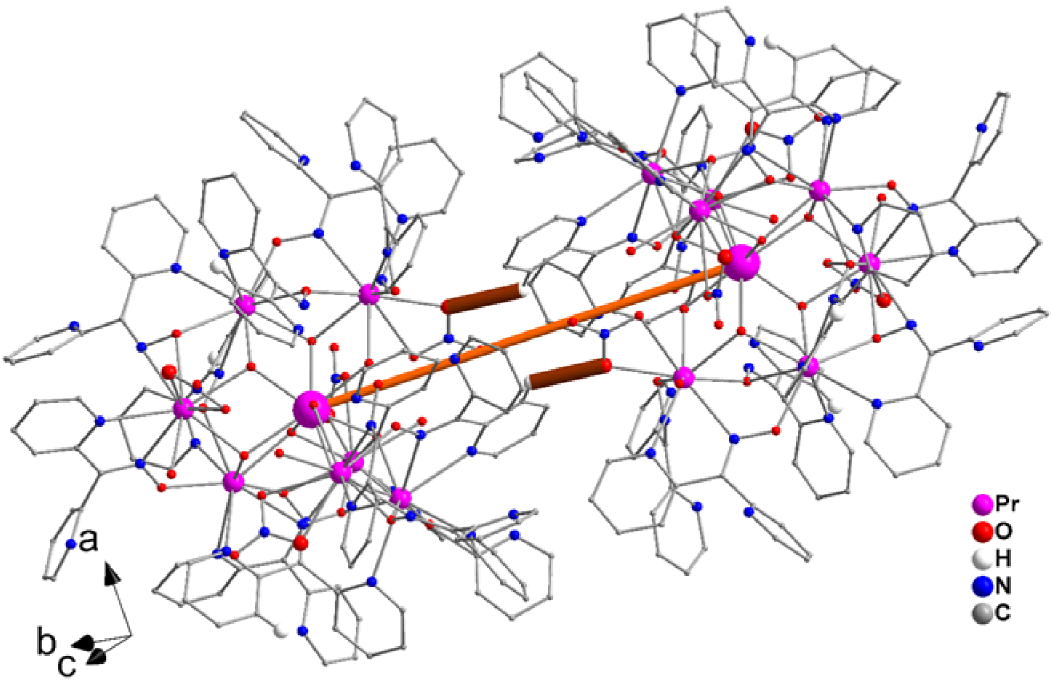

a)

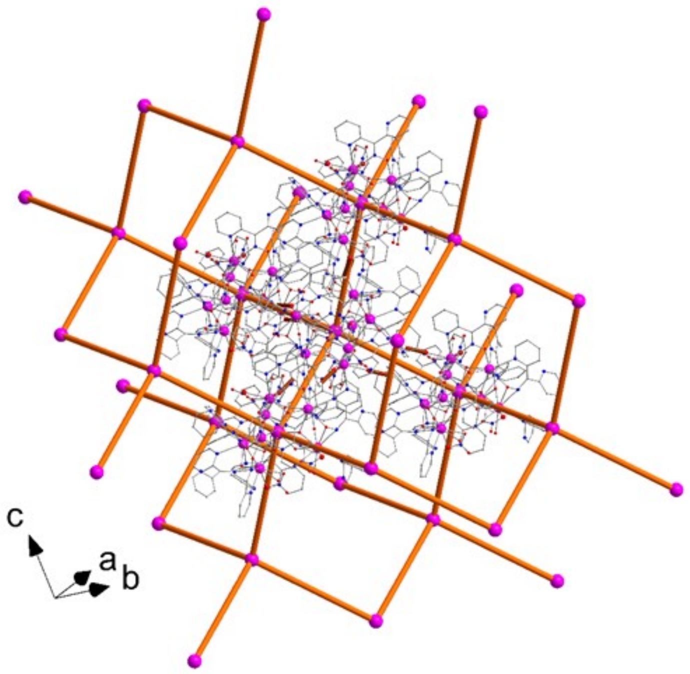

b)

Figure S21. (a) A pair of molecules **4** interacting through a $C_{pyridyl}$-H···$O_{coordinated\ nitrato}$ H-bonding interaction (indicated with thick brown lines). (b) Through these interactions, the neighboring complex molecules form a diamond-like lattice. The thick orange lines are guides to the eye to show the arrangement of neighboring cluster molecules in the lattice of the structure.

Short Description of the Supramolecular Characteristics in the Crystal Structure of 1·3MeCN

The dinuclear molecules **1** from "dimers" through the H-bonding interactions C4-H4···O66 and C2-H2···O75 (Table S3) and π-π interactions between the centrosymmetrically-related planar rings N1C1···C5 and N1'C1'···C5' (indicated with dashed green lines in Figure S9; symmetry code: '1-*x*, -*y*, 2-*z*) at a distance of 3.358(8) Å between the planes (Figure S9). Interdimer π-π stacking interactions between the centrosymmetrically-related planar rings N33C43···C47 and N33''C43''···C47'' (indicated with dashed light green lines in Figure S9; symmetry code: ''1-*x*, -*y*, 1-*z*), at a distance of 3.48(4) Å, result in the formation of chains of molecules **1** parallel to the *c* axis. Through C37-H37···O73 interactions (Table S3), indicated with dashed cyan lines in Figure S10, the molecules form layers parallel to the (1-10) planes. The layers further interact through C49-H49···O63 (Figure S11, Table S3) H bonds to build the 3D architecture. When a portion of the structure is viewed along the –*a* crystallographic direction, voids are seen in which the lattice MeCN molecules are hosted (Figure S11). The lattice solvent molecules, hosted in the lattice, are engaged in H-bonding interactions with the complex molecules **1** (Table S3).

Short Description of the Supramolecular Characteristics in the Crystal Structure of 2·3MeNO$_2$

The dinuclear molecules from layers parallel to the (001) planes (Figure S3) through C43-H43···O69 and C15-H15···O63 non-classical H bonds. Molecules belonging to neighboring layers are stacked along the *c* axis interacting through C2-H2···O69 (symmetry operation: 2-*x*, 1-*y*, -*z*) H-bonding interactions. The molecules are also connected through π-π stacking interactions between the symmetrically-related planar rings N1C1···C5 and N1'C1'···C5' (indicated with dashed pink lines in Figure S4; symmetry code: '2-*x*, 1-*y*, -*z*) at a distance of 3.26(3) Å between the planes, and the centrosymetrically-related planar rings N35C48···C52 and N35''C48''···C52'' (indicated with dashed green lines in Figure S4; symmetry code: ''2-*x*, -*y*, 1-*z*) at a distance of 3.43(4) Å between the planes. Through these C2-H2···O69 and π-π stacking interactions, the 3D architecture of the structure is formed. In the structural view shown in Figure S4, voids are clearly seen; the voids host lattice MeNO$_2$ molecules which interact with the complex molecules through weak C-H···O bonds, forming the framework of the structure. Details for the H-bonding interactions are provided in Table S2.

Short Description of the Supramolecular Characteristics in the Crystal Structure of 3·2MeCN

The tetranuclear molecules **3** form chains along the *a* axis (Figure S15) through π-π interactions between the centrosymmetrically-related planar aromatic rings N1C1···C5 and N1''C1''···C5'' (indicated with dashed dark green lines in Figure S15; symmetry code: ''3-*x*, 1-*y*, 1-*z*) at a distance of 3.41(5) Å between the planes. Through C10-H10···O33 H-bonding interactions, the chains form layers of the molecules parallel to the (010) planes (Figure S16, Table S4). Molecules belonging to neighboring layers interact through C31-H31···N13 and C50-H50···O43 H-bonding interactions contributing to the formation of the 3D architecture of the structure (Figure S17, Table S4). Voids formed in the crystal structure host a lattice MeCN molecule (Figure S17) and interact with the 3D framework through H bonds (Table S4).

Very Short Description of the Supramolecular Characteristics in the Crystal Structure of 4·4EtOH·4(n-hexane)

Neighboring nonanuclear molecules interact through $C_{pyridyl}$-H···$O_{coordinated\ nitrato}$ H-bonding interactions (Figure S21a) and through this interaction a diamond-like lattice is formed (Figure S21b). The dimension of this interaction (intermolecular cells) are: C4-H4-O62 (3/4+*y*, ¾-*x*, -1/4+*z*), C4···O62 3.101(8) Å, H4···O2 2.45 Å, C4-H4···O2 = 126°.

Mechanistic Proposals for the Formation of the Key-Intermediate VI Which Leads to Coordinated L$^-$ in Complexes 1·3MeCN and 2·3MeNO$_2$

The proposed mechanism for the Pr(III)-mediated hydrolysis of dpkoxH in the absence of Et_3N to produce the key-intermediate **VI** is illustrated in Scheme S1.

Scheme S1. Proposed Mechanism for the Pr(III)-Mediated Hydrolysis of dpkoxH in the Absence of Et_3N to Produce the Key Intermediate VI^{a,b}

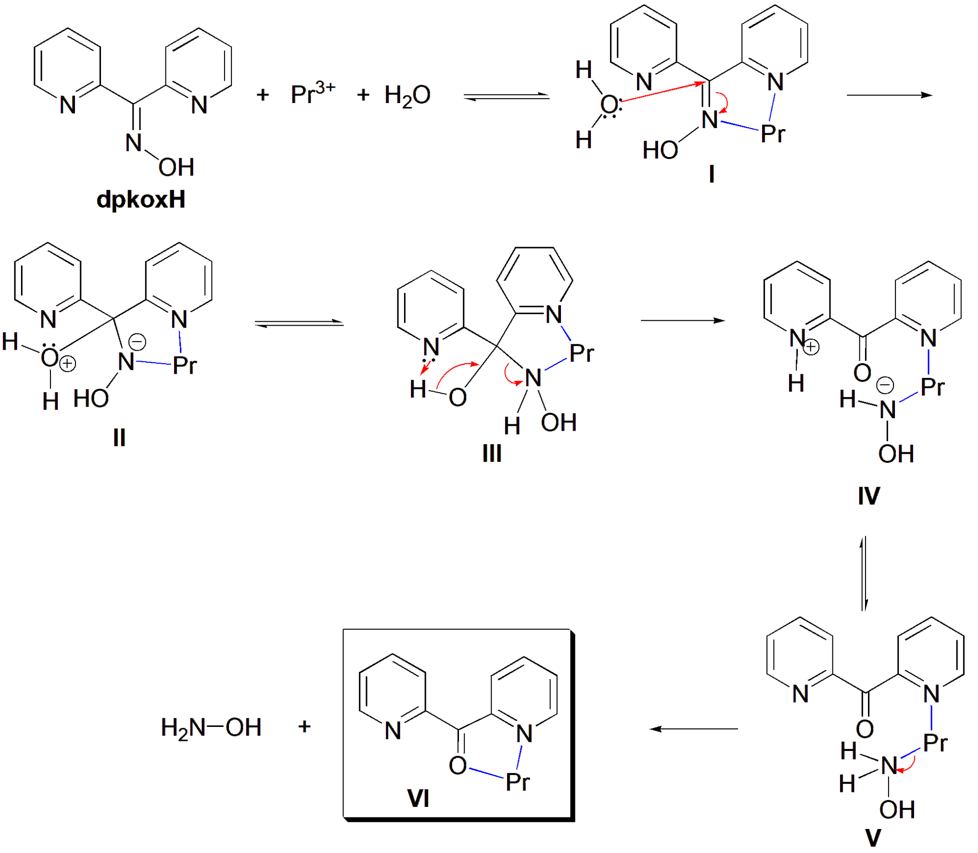

^{a} The nitrato ligands and (possibly) coordinated solvent molecules have been omitted for clarity. ^{b} Further reactions of **VI** to give the coordinated L$^-$ ligand are illustrated in Scheme 3 of the main body of the paper.

The mechanistic Scheme S1 involves the following steps: (i) Complexation of Pr^{3+} through the N atom of one of the 2-pyridyl rings and the N atom of the oxime functionality, with the formation of a 5-membered chelating ring, leads to species **I**. The complexation results in amplification of the electrophilicity of the oxime C atom. (ii) The weak nucleophile H_2O can then attack this atom resulting in the tetrahedral intermediate **II**. (iii) An O to N proton transfer then follows leading to the new intermediate **III**. (iv) This collapses to the intermediate **IV**. (v) A N to N proton transfer gives rise to the new intermediate **V**, and finally (vi) **V** liberates H_2NOH and the chelate species **VI** is formed, in which Pr^{III} is ligated by the O atom of the uncovered carbonyl function and the N atom of a 2-pyridyl ring through a 5-membered chelating ring; this coordination mode of dpk has been crystallographically observed in lanthanoid(III) chemistry.[1]

The proposed mechanism for the Pr(III)-mediated hydrolysis of dpkoxH in the presence of Et_3N to produce the key-intermediate **VI** is illustrated in Scheme S2.

Scheme S2. Proposed Mechanism for the Pr(III)-Mediated Hydrolysis of dpkoxH in the Absence of Et_3N to Produce the Key Intermediate VI[a,b]

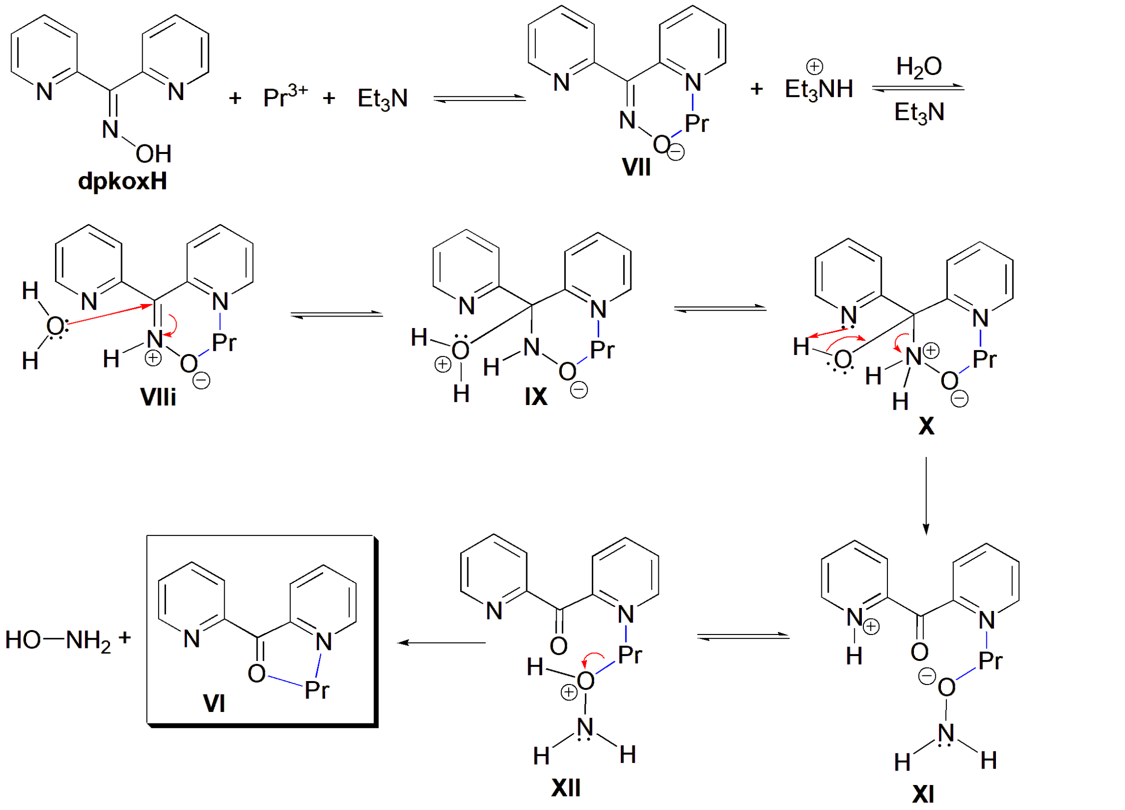

[a] The nitrato ligands and (possibly) coordinated solvent molecules have been omitted for clarity. [b] Further reactions of **VI** to give the coordinated L^- ligand are illustrated in Scheme 3 of the main body of the paper.

The mechanistic Scheme S2 involves the following steps: (i) Complexation of Pr^{3+} through the N atom of one 2-pyridyl ring and the deprotonated O atom of the oxime functionality, with the formation of a 6-membered chelating ring, leads to species **VII**. (ii) Proton exchange between the triethylammonium cation and the N atom of the oxime functionality results in amplification of the electrophilicity of the oxime C atom (intermediate **VIII**). (iii) The weak nucleophile H_2O can then attack this atom resulting in the tetrahedral intermediate **IX**. (iv) An O to N proton transfer generates the new intermediate **X**. (v) This collapses to the intermediate **XI**. (vi) A N to O proton transfer leads to the new intermediate **XII**, and finally (vii) **XII** liberates H_2NOH and the new chelate species **VI**.

REFERENCE

(1) Rey, P.; Caneschi, A.; Sukhikh, T. S.; Vostrikova, K. E. Tripodal Oxazolidine-N-Oxide Diradical Complexes of Dy^{3+} and Eu^{3+}. *Inorganics* **2021**, *9*, article 91.
